# Endothelial cell-derived RSPO3 activates Gαi1/3-Erk signaling and protects neurons from ischemia/reperfusion injury

**DOI:** 10.1038/s41419-023-06176-2

**Published:** 2023-10-07

**Authors:** Ting-tao Liu, Xin Shi, Hong-wei Hu, Ju-ping Chen, Qin Jiang, Yun-Fang Zhen, Cong Cao, Xue-wu Liu, Jian-gang Liu

**Affiliations:** 1 Shandong University, Department of Neurology, Shandong Provincial Hospital, Jinan, China; 2 Department of Neurology, Shouguang Hospital of T.C.M, Shouguang, China; 3 Department of Neurology and Clinical Research Center of Neurological Disease, The Second Affiliated Hospital of Soochow University, Suzhou, China; 4 Department of Neurosurgery, The First Affiliated Hospital of Soochow University, Suzhou, China; 5 Department of Neurology, Changshu Hospital of Traditional Chinese Medicine, Changshu, China; 6 The Fourth School of Clinical Medicine, Nanjing Medical University, Nanjing, China; 7 Department of Orthopedics, Children’s hospital of Soochow University, Suzhou, China; 8 Department of Neurology, Shandong Provincial Hospital Affiliated to Shandong First Medical University, Jinan, China

**Keywords:** Apoptosis, Growth factor signalling

## Abstract

The current study explores the potential function and the underlying mechanisms of endothelial cell-derived R-spondin 3 (RSPO3) neuroprotection against ischemia/reperfusion-induced neuronal cell injury. In both neuronal cells (Neuro-2a) and primary murine cortical neurons, pretreatment with RSPO3 ameliorated oxygen and glucose deprivation (OGD)/re-oxygenation (OGD/R)-induced neuronal cell death and oxidative injury. In neurons RSPO3 activated the Akt, Erk and β-Catenin signaling cascade, but only Erk inhibitors reversed RSPO3-induced neuroprotection against OGD/R. In mouse embryonic fibroblasts (MEFs) and neuronal cells, RSPO3-induced LGR4-Gab1-Gαi1/3 association was required for Erk activation, and either silencing or knockout of Gαi1 and Gαi3 abolished RSPO3-induced neuroprotection. In mice, middle cerebral artery occlusion (MCAO) increased RSPO3 expression and Erk activation in ischemic penumbra brain tissues. Endothelial knockdown or knockout of RSPO3 inhibited Erk activation in the ischemic penumbra brain tissues and increased MCAO-induced cerebral ischemic injury in mice. Conversely, endothelial overexpression of RSPO3 ameliorated MCAO-induced cerebral ischemic injury. We conclude that RSPO3 activates Gαi1/3-Erk signaling to protect neuronal cells from ischemia/reperfusion injury.

## Introduction

Cerebral infarction accounts for over 75% of acute cerebrovascular disease [1, 2]. Damage or occlusion of blood vessels results in ischemic and hypoxic injury in the ischemic area and adjacent brain tissues [3, 4]. Cerebral ischemia-reperfusion injury will aggregate brain injury [1, 5], causing mitochondrial dysfunction, oxidative injury, Ca^2+^ overload, blood brain barrier damage, inflammation and nitric oxide toxicity, eventually leading to apoptotic death of neurons [6, 7]. Oxygen and glucose deprivation (OGD)/re-oxygenation (OGD/R) [8] model mimics ischemia/reperfusion injury to cultured neurons (and neuronal cells) [9–12].

The R-spondin (RSPO) family of proteins include four primary members, including R-spondin1 (RSPO1), R-spondin2 (RSPO2), R-spondin3 (RSPO3), and R-spondin4 (RSPO4) [13]. RSPOs have similar protein structures consisting of two amino-terminal furin-like (FU1-FU2) repeats, a thrombospondin (TSP) domain and a basic amino acid-rich carboxy-terminal domain (BR) [13], but perform markedly different functions [13–15]. The FU1, FU2 and TSP/BR domains connect RSPO proteins with the ubiquitin ligase ZNRF3 (Zinc And Ring Finger 3)/RNF43 (Ring Finger Protein 43), leucine-rich repeat G protein-coupled receptor 4–6 (LGR4–6) and heparin sulfate proteoglycan (HSPG) [16–23].

RSPO3 has been shown to play an essential role in vascular development and angiogenesis [24, 25]. Conditional RSPO3 knockout (KO) in the developing heart resulted in defective development of the secondary cardiac field, showing pericardial edema and marked vascular congestion [26]. Scholz et al. found that depletion of endothelial-derived RSPO3 was the primary cause of the death of RSPO3 KO mice [24]. RSPO3 in endothelial cells was shown to regulate vascular remodeling, inhibit endothelial cell apoptosis [24], and promote coronary formation in angiogenic regions of the heart [27].

Recent studies report that RSPO3 activates Wnt signaling to promote stem cell recovery and epithelial repair [28]. Contrarily, stromal depletion of RSPO3 impedes regeneration of crypt [28]. Sigal et al. demonstrated that RSPO3 induces the differentiation of basal Lgr5^+^ cells into secretory cells, inhibiting *H. pylori* colonization in gastric glands through the secretion of intelectin-1 and other antimicrobial factors [29]. Zhou et al. showed that endothelial KO of RSPO3 suppressed anti-inflammatory interstitial macrophage formation and caused severe inflammatory response in endotoxemic mice [30]. Here we examined the potential function and the underlying mechanisms of endothelial cell-derived RSPO3 against ischemia/reperfusion-induced neuronal cell injury.

## Materials and methods

### Reagents

As described in our previous study [31], the recombinant human RSPO3 was purchased from Sigma-Aldrich (SRP3323), and both human (human umbilical vein endothelial cells/HUVECs) and murine cells (mouse embryo fibroblasts/MEFs) were response to it [31]. Puromycin, polybrene, PD98059 and U0126 were purchased from Sigma-Aldrich. Fetal bovine serum (FBS), medium, antibiotics and other cell culturing reagents were purchased from Gibco Co. (Shanghai, China). Antibodies utilized in this study were described in our previous studies [31–34] or otherwise mentioned. The primers, sequences and viral constructs were obtained from Genechem Co. (Shanghai, China), unless otherwise mentioned. The recombinant RSPO3 protein was first dissolved in PBS at 50 μg/mL as the stock solution, which was then added directly to medium of the neuronal cells/neurons.

### Cells

The wild-type (WT) mouse embryonic fibroblasts (MEFs), the Gαi1 and Gαi3 double knockout (DKO) MEFs, Gαi1, Gαi2, or Gαi3 single knockout (SKO) MEFs, WT and Gab1 knockout (KO) MEFs were described in our previous studies [31–34]. Neuro-2a cells were purchased from the Cell Bank of CAS Shanghai Institution of Biological Science (Shanghai, China). Expression of Gαi1, Gαi2, and Gαi3 were always checked [33–37]. For isolation and primary culture of murine cortical neurons, fronto-lateral cortical lobes from embryonic day 15.5 (E15.5) C57BL/6 mice fetuses were dissected. Afterwards, brain cells were chemically dissociated by adding trypsin and DNase I (Sigma-Aldrich) and were resuspended in serum-free B27 Neurobasal medium (Gibco) plus penicillin, streptomycin and L-glutamine (Gibco). Cells were placed on poly-L-lysine (Sigma Aldrich)-coated culture plates or glass coverslips and maintained at 37 °C in a saturated atmosphere with 95% air and 5% CO_2_. At day-10 (DIV), 91.52 ± 3.57% of cells were primary murine cortical neurons. The purity was calculated by measuring NeuN-positive nuclei percentage. Newborn mice (C57BL/6 J) were sacrificed at postnatal day 1 (P1). The brain cortices were dissected and cut into small pieces, and primary cells dissociated with trypsin. The cells were then strained through 70 μm cell strainer and plated in culture flasks coated with poly-D-lysine. Mixed glial cells were cultured for 7–9 days. The culture flask was then placed vertically for 5 min, then the culture bottle is gently patted for 10–15 min by hand, and the separation of microglia is observed under the microscope every 5 min. At this time, the microglia growing in the upper layer fell off into the culture medium. Microglia were collected, centrifuged at 1200 rpm for 5 min, resuspended and seeded onto poly-L-lysine pre-coated culture dish/flasks. Microglia were verified by positive staining of Iba-1 (over 95%-positive, Abcam). After collecting microglia, the culture flask was shaken at 250 rpm for 18 h to remove non-astrocytes. Astrocyte phenotype was evaluated by cell exhibiting a characteristic morphology and positive staining for the astrocytic marker glial fibrillary acid protein (GFAP, over 95%-positive, Abcam). The protocols of the present study were approved by the Ethics Committee and Institutional Animal Care and Use Committee (IACUC) of Soochow University (2019BR0151).

### Isolation and primary culturing of mouse brain endothelial cells

The detailed protocols were described previously [38]. Briefly, C57BL6J mice were sacrificed. The brain cortices were dissected and cut into small pieces. Tissues were then subject to two rounds of enzymatic dissociation and myelin separation as described [38]. FBS was then added to block the trypsin/collagenase activity. The solution was then filtered through a 10 nm sterile mesh adapted to a 50 ml conical tube. The mesh was further washed with medium to recover more cells and was centrifuged at 800 × g for 15 min at room temperature with the supernatant discarded. The pellet was then cultured in described 20% FBS-containing DMEM/F-12 medium plus human basic fibroblast growth factor (bFGF) and bovine sodium heparin [38]. Medium was renewed every 48 h. The passage of primary endothelial cells was carried out after seven days of culture and endothelial cells enrichment was obtained through passaging as described [38]. The purity of the endothelial cells, 94.03 ± 4.16%, was calculated by measuring the percentage of cells with positive vascular cell adhesion molecule 1 (VCAM-1) fluorescence staining.

### OGD/re-oxygenation (OGD/R)

OGD/R procedure was described previously [9, 39]. Neuronal cells, primary murine neurons or mouse brain endothelial cells, with the applied genetic modifications or treatments, were cultivated in glucose-free medium and were placed in a sealed airtight chamber with continuous flux of gas (95% N_2_/5% CO_2_) for 4 h (OGD). Cells were then re-oxygenated (OGD/R) for designated time periods. Neuronal cells in the norm-oxygenated medium containing glucose were labeled as “Mock” control cells.

### Lipid peroxidation assays

Neuronal cells were seeded into the poly-L-lysine-coated six-well plates at 120,000 cells per well. Following the designated treatment, the thiobarbituric acid reactive substances (TBAR)-reactive malondialdehyde (MDA) concentration was measured. The latter reacts with the thiobarbituric acid and forms the pink complex. The reaction buffer [40, 41] was mixed with cell lysates and the mix was boiled at 100 °C for 30 min. Thereafter, the mix was centrifuged at 3500 rpm for 12 min and the absorbance of pink color was measured at 535 nm.

### Genetic modifications in vitro

Gαi1 and Gαi3 double silencing by targeted lentiviral shRNA, ectopic Gαi1 and Gαi3 overexpression by adenoviral constructs, CRISPR/Cas9-induced Gαi1 and Gαi3 double knockout (DKO), rescuing Gαi1 or Gαi3 in Gαi1/3 DKO MEFs, the dominant negative (DN)-Gαi1 or DN-Gαi3, or the empty vector (“Vec”), were reported in our previous studies [31–37, 42]. The GV369 constructs containing the LGR4 shRNA (sh-LGR4-s1 or sh-LGR4-s2), Gab1 shRNA or Gab1 cDNA were provided by Genechem (Shanghai, China), each was transduced to HEK-293 cells by Lipofectamine 3000 to generate lentivirus. Virus were thereafter enriched, purified and quantified, and were added (at MOI = 15) to cultured cells/neurons. Stable cells were formed after selection using puromycin-containing medium.

### Fluorescence dye assays in vitro

The detailed protocols have been described in our previous studies [43, 44]. Neuronal cells or neurons were placed onto poly-L-lysine pre-coated coverslips at 60–70% confluence and were subject to the designated treatments. Cells were then fixed by using 4% formaldehyde solution for 12 min and were permeabilized by Triton X-100 solution (0.2%) for additional 7.5 min at room temperature. Cells were then stained with the applied fluorescence dyes (JC-1, CellROX, TUNEL and DAPI). Fluorescence images were taken through the fluorescence microscopy (Zeiss) and the fluorescence intensity was measured from five random views of each treatment.

### Other assays

The detailed protocols of Western blotting, co-immunoprecipitation (Co-IP), quantitative reverse transcription PCR (qRT-PCR), cell counting kit-8 (CCK-8), medium lactate dehydrogenase (LDH) releasing, Caspase-3/−9 activity assay, as well as single strand DNA (ssDNA) ELISA and Histone DNA ELISA are widely utilized in our previous studies [43, 45–49]. Figure S4 listed the uncropped blotting images.

### The middle cerebral artery occlusion (MCAO) model and 2,3,5-triphenyltetrazolium hydrochloride (TTC) staining

Mice (all male, 9–10 week old, 21–22 g), from Changzhou Cavens Experimental Animal Co (Changzhou, China), were anesthetized with isoflurane (3% for induction and 1.5% during the rest of the surgical procedure under 30% oxygen and 70% nitrogen). A silicon-coated 6–0 nylonsuture was introduced into the external carotid artery and advanced up to the internal carotid artery to occlude the middle cerebral artery for 1 h followed by reperfusion. The rectal temperature of the mice was always maintained at 37.0 °C using a temperature-control heating pad. Animal regional cerebral blood flow (rCBF) was monitored via a transcranial laser Doppler, and mice with the rCBF higher than 20% of original baseline value were excluded. The nylon suture was removed 1 h after common carotid artery occlusion, and the skin incision was theater closed. The “Mock” group mice underwent the same surgical procedures but without MCAO. Twenty-four hours after reperfusion, the brain was collected, sectioned and stained with TTC (1%, at 37 °C for 1 h). Afterwards, the brain sections were fixed and photographed. The infarction area ratio = (contralateral hemispheric volume - ipsilateral non-infarct volume)/contralateral hemispheric volume × 100%. The brain area of each section was measured using the National Institutes of Health Image program (Image J 1.37 v). The animal protocols were conducted in according to the Institutional Animal Care and Use Committee and the Ethic Committee of Soochow University (2019BR0151).

### Immunohistofluorescence

Mice were anaesthetized and underwent intracardial perfusion with 0.9% saline followed by 4% paraformaldehyde. Brains were post-fixed in paraformaldehyde (4%) for 24 h at 4 °C and then maintained under 30% sucrose. Brains were sectioned with a sliding-freezing microtome (Leica). Mouse brain sections were incubated with blocking buffer (0.4% Triton X-100 and 5% goat serum) for 1 h. Brain sections were incubated with primary antibody overnight at 4 °C, washed 3 times in PBS, then incubated with secondary antibodies for 2 h at room temperature. The following primary antibodies were utilized: rabbit anti-Erk1/2 (phospho Thr202/Tyr204) (Cell Signaling Technology, 4370 s, 1:100), mouse anti-NeuN (Cell Signaling Technology, 94403 S, 1:100), rabbit anti-RSPO3 (Proteintech, 17193-1-AP, 1:100). All secondary antibodies were used at a concentration of 1:500. Secondary antibodies were goat anti-rabbit Alexa 488 (Abcam, ab150077) and goat anti-mouse Alexa 594 (Abcam, ab150116).

### Neurological (Garcia) scores

As reported [50], 24 h after reperfusion, the neurological functions of MCAO mice and Mock mice were measured. Six grades were involved in Garcia scores containing spontaneous activity, spontaneous movements of all limbs, movements of forelimbs, climbing wall of wire cage, touch of trunk and Vibrissae touch. The minimum score 0 for each grade is severest deficit, and the maximum score is 3 (normal) [50]. The total score is 18 (healthy), and the lower score indicated serious deficits [50].

### The foot-fault test

For testing of motor coordination in MCAO mice and Mock mice, foot-fault tests were performed 14 days after MCAO using the described protocol [51]. In brief, mice were placed on a horizontal grid floor and were allowed to walk for 2 min. A foot fault was recorded when the mouse’s foot miss–stepped on the grid and the foot fell downwards through the opening between the grids. All four limbs were observed for misses. The percentage of total foot faults was recorded [51].

### Genetic modifications in vivo

RSPO3 shRNA) or RSPO3 cDNA sequence [NM_028351.3] was inserted into an adeno-associated virus 5 (AAV5)-TIE1 construct (reported in our previous studies [31, 32, 42, 52]) that contained sequence of the endothelial specific promoter TIE1 [52]. The constructs were individually transfected to HEK-293 cells to generate adenovirus, which was intravitreally injected to the mice as reported (at 2 μL virus per mouse, 0.2 μL per minute) [52]. The TIE1-DIO-Cre C57 mice were commercial available and were reported previously [52]. The AAV5 construct encoding FLEX plus the dCas9-murine *RSPO3* sgRNA sequence, or AAV5-FLEX-CRISPR/Cas9-RSPO3-KO, was designed by Genechem (Shanghai, China), and AAV generated and was intravitreally injected to TIE1-DIO-Cre C57 mice (at 2 μL virus per mouse, 0.2 μL per minute). For infection of primary neurons or endothelial cells, the RSPO3 shRNA or RSPO3 cDNA were inserted into the GV369 construct containing the TIE1 promoter sequence, which was then transduced to HEK-293 cells to generate lentivirus. Lentivirus was added to the cells for 96 h.

### Statistical analysis

**Fig. 1:**
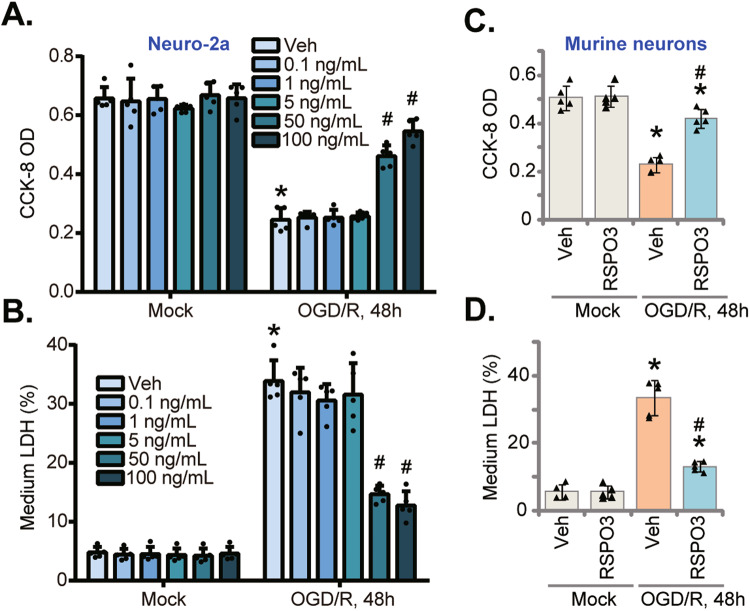
RSPO3 ameliorates OGD/R-induced neuronal cell death. Neuro-2a neuronal cells (**A**, **B**), the primary murine cortical neurons (**C**, **D**) were pretreated with RSPO3 (at 50 ng/mL, expert for **A**, **B**) or the vehicle control (“Veh”) for 30 min, followed by oxygen glucose deprivation (OGD) for 4 h and then re-oxygenation (OGD/R) for 48 h, viability and cell death were tested by CCK-8 (**A**, **C**) and medium LDH releasing (**B**, **D**) assays, respectively. “Mock” stands for the mock treatment (norm-oxygenated medium with glucose). Data were presented as mean ± standard deviation (SD, *n* = 5). *P < 0.001 *vs*. “Mock” cells with Veh treatment. ^#^*P* < 0.001 *vs*. OGD/R with “Veh” pretreatment. Each experiment was repeated five times and similar results were obtained.

Data in this study were all with normal distribution and were presented as mean ± standard deviation (SD). The two-tailed unpaired *t*-test was utilized to compare statistical difference between two specific groups. One-way analysis of variance (ANOVA) plus Tukey’s post hoc tests were utilized for multiple groups (unless otherwise mentioned in Fig. 1). *P* < 0.05 was statistically significant.

## Results

### RSPO3 ameliorates OGD/R-induced neuronal cell death

To test the potential effect of RSPO3 on ischemia/reperfusion-caused neuronal damage, in vitro experiments were carried out on Neuro-2a neuronal cells [53, 54]. Neuro-2a cells were maintained under oxygen glucose deprivation (OGD) for 4 h and then cultured in the complete medium (“re-oxygenation”, OGD/R) for another 48 h. OGD/R decreased CCK-8 viability (Fig. 1A) and provoked Neuro-2a cell death as evidenced by increased LDH release (Fig. 1B).

Testing whether exogenously-added RSPO3 was neuroprotective, pretreatment with RSPO3 was found to inhibit OGD/R-induced cytotoxicity in Neuro-2a cells (Fig. 1A, B). The RSPO3-induced neuronal protection was dose-dependent and was significant at 50 and 100 ng/mL (Fig. 1A, B). Treatment with RSPO3 alone at the tested concentrations failed to significantly alter CCK-8 viability (Fig. 1A) and LDH release (Fig. 1B). Although higher concentrations of RSPO3 inhibited the reduction in OGD/R-induced Neuro-2a cell viability (Fig. S1A) and LDH medium release (Fig. S1B), the 50–100 ng/mL concentration exhibited better neuroprotection (Fig. S1A, B). Titration results revealed that RSPO3, at 50 ng/mL, robustly inhibited OGD/R-induced neuronal cell death, and this concentration was selected for the following studies. We also found that pretreatment of RSPO3 for 15′-60′ significantly inhibited OGD/R-induced cytotoxicity in Neuro-2a cells (Fig. S1C, D), with 30′ of pretreatment showing the most significant neuroprotective effects (Fig. S1A, B).

Testing RSPO3 in primary neurons, OGD/R decreased cell viability in primary murine cortical neurons (Fig. 1C) and increased LDH release (Fig. 1D), which were ameliorated byRSPO3 (50 ng/mL) pretreatment (Fig. 1C, D).

### RSPO3 ameliorates OGD/R-induced neuronal cell apoptosis

**Fig. 2:**
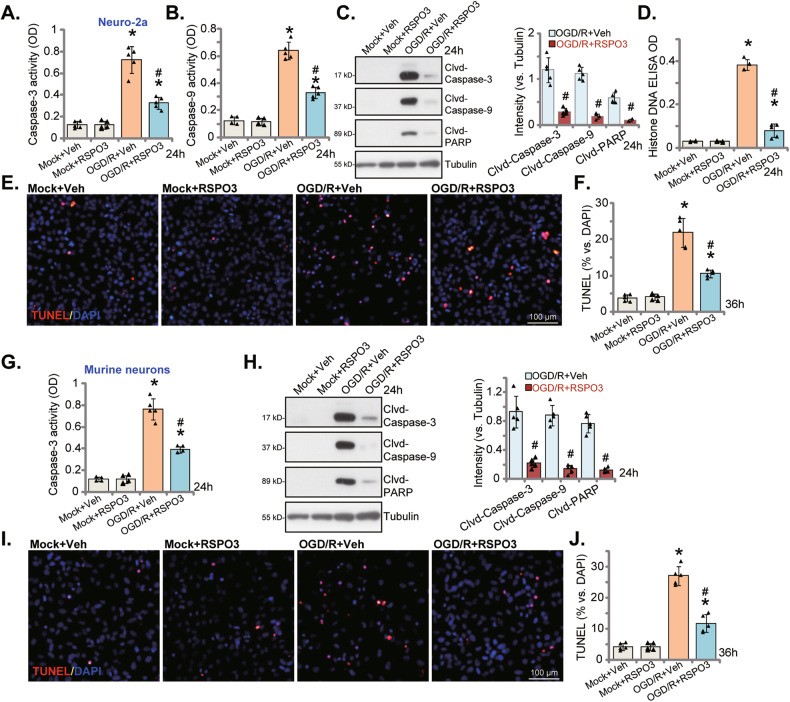
RSPO3 ameliorates OGD/R-induced neuronal cell apoptosis. Neuro-2a neuronal cells (**A**–**F**) or the primary murine cortical neurons (**G**–**I**) were pretreated with RSPO3 (at 50 ng/mL) or the vehicle control (“Veh”) for 30 min, followed by oxygen glucose deprivation (OGD) for 4 h and then re-oxygenation (OGD/R) for applied time periods, the caspase-3/9 activities (**A**, **B**, **G**) were tested; Expression of listed proteins in total cell lysates were shown (**C**, **H**); Histone-bound DNA contents were measured (**D**). Cell apoptosis was examined by TUNEL staining (**E**, **F**, **I**, **J**). Data were presented as mean ± standard deviation (SD, *n* = 5). *P < 0.001 *vs*. “Mock” cells. ^#^*P* < 0.001 *vs*. OGD/R with “Veh” pretreatment. Each experiment was repeated five times and similar results were obtained. Scale bar = 100 μm.

Apoptosis is the primary mechanism of OGD/R-induced neuronal cytotoxicity [55–58]. Following OGD/R stimulation, the relative caspase-3 activity (Fig. 2A) and the relative caspase-9 activity (Fig. 2B) were significantly increased in Neuro-2a cells. As shown in Fig. 2C, OGD/R treatment led to cleavage of caspase-3, caspase-9 and poly (ADP-ribose) polymerase (PARP), and histone-bound DNA counts were increased in OGD/R-stimulated Neuro-2a cells (Fig. 2D). Remarkably, RSPO3 (at 50 ng/mL) pretreatment largely attenuated OGD/R-induced apoptosis in Neuro-2a cells (Fig. 2A–D), and decreased the percentage of TUNEL-positively stained nuclei (Fig. 2E, F). In primary murine cortical neurons, RSPO3 (at 50 ng/mL) pretreatment ameliorated OGD/R-induced caspase-3 activation (Fig. 2G), caspase-3/caspase-9/PARP cleavage (Fig. 2H) and decreased TUNEL-positively stained nuclei ratio (Fig. 2I, J).

### RSPO3 ameliorates OGD/R-induced oxidative injury in neuronal cells

**Fig. 3:**
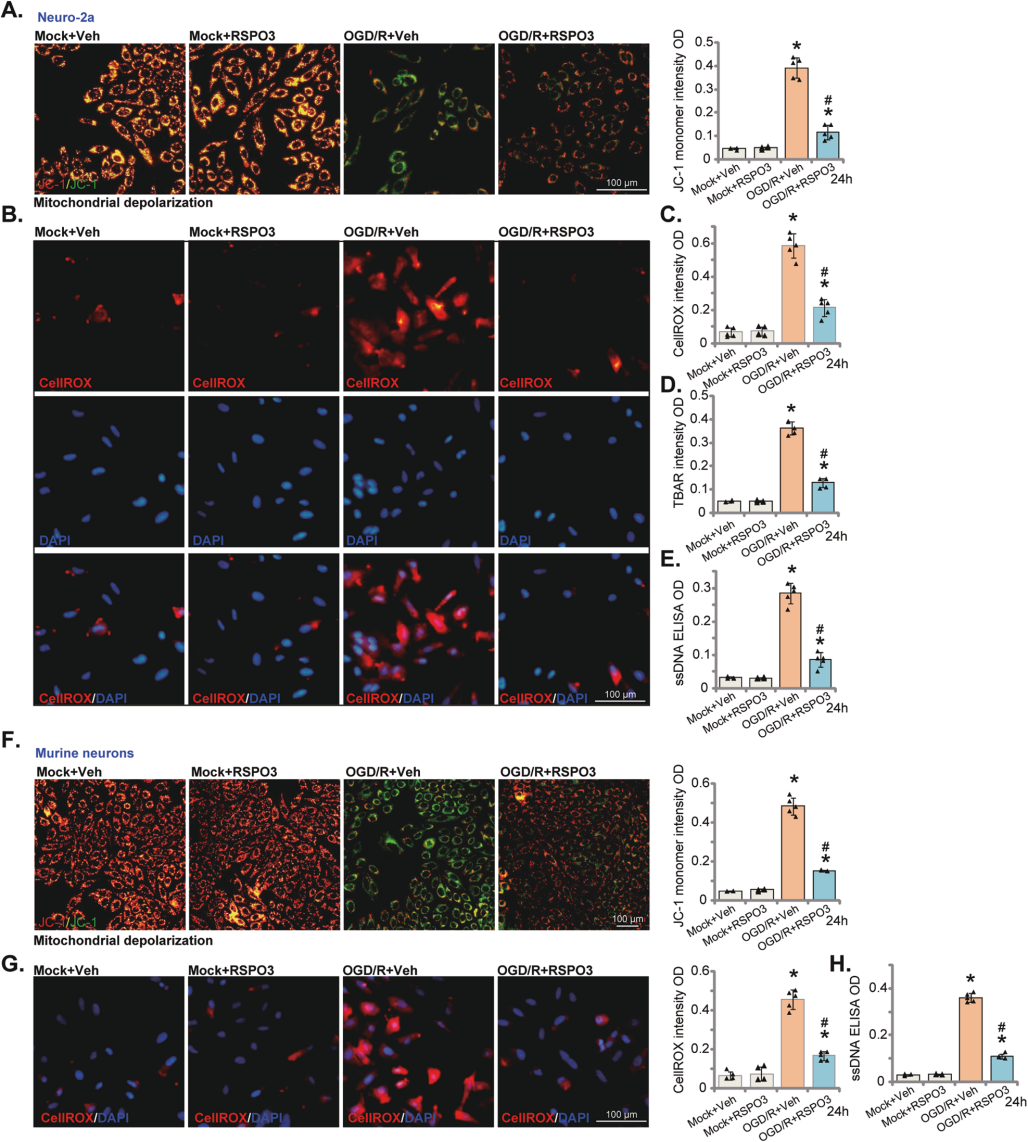
RSPO3 ameliorates OGD/R-induced oxidative injury in neuronal cells. Neuro-2a neuronal cells (**A**–**E**) or the primary murine cortical neurons (**F**–**H**) were pretreated with RSPO3 (at 50 ng/mL) or the vehicle control (“Veh”) for 30 min, followed by oxygen glucose deprivation (OGD) for 4 h and then re-oxygenation (OGD/R) for 24 h, mitochondrial depolarization (**A**, **F**), ROS production (**B**, **C**, **G**), lipid peroxidation (**D**) and ssDNA contents (**E**, **H**) were tested by the described assays. “Mock” stands for the mock treatment (norm-oxygenated medium with glucose). Data were presented as mean ± standard deviation (SD, *n* = 5). *P < 0.001 *vs*. “Mock +Veh” group. ^#^*P* < 0.001 *vs*. OGD/R with “Veh” pretreatment. Each experiment was repeated five times and similar results were obtained. Scale bar = 100 μm.

OGD/R disrupts mitochondrial function, causing ROS production and oxidative injury, serving as a primary cause of neuronal cell death/apoptosis [8–10, 59–61]. In Neuro-2a cells, OGD/R caused mitochondrial depolarization (Fig. 3A), indicated by the conversion of JC-1 red fluorescence to green fluorescence (monomers, Fig. 3A). ROS production was increased as shown by an increase in CellROX red fluorescence intensity (Fig. 3B, C) in OGD/R-stimulated Neuro-2a neuronal cells. Pretreatment with RSPO3 (50 ng/mL) inhibited OGD/R-induced mitochondrial depolarization (Fig. 3A) and ROS production (Fig. 3B, C) in Neuro-2a cells. OGD/R caused lipid peroxidation (TBAR intensity increasing, Fig. 3D) and DNA breaks (ssDNA accumulation, Fig. 3E). Both were significantly ameliorated after RSPO3 pretreatment (Fig. 3D, E).

In the primary murine cortical neurons, OGD/R similarly provoked mitochondrial depolarization (JC-1 green monomer accumulation, Fig. 3F), ROS production (CellROX, Fig. 3G) and DNA breaks (ssDNA accumulation, Fig. 3H). These actions were ameliorated following RSPO3 pretreatment (Fig. 3F–H), demonstrating that RSPO3 can efficiently ameliorate OGD/R-induced oxidative injury in neuronal cells.

### RSPO3-induced neuroprotection against OGD/R is abolished after Erk1/2 inhibition

**Fig. 4:**
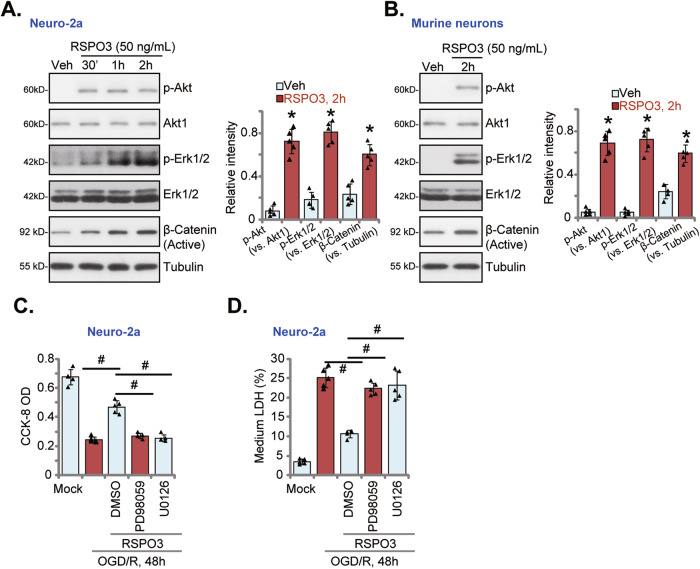
RSPO3-induced neuroprotection against OGD/R is abolished after Erk1/2 inhibition. Neuro-2a neuronal cells (**A**) or the primary murine cortical neurons (**B**) were treated with RSPO3 (at 50 ng/mL) or the vehicle control (“Veh”) for indicated time periods, and expression of listed proteins was shown. Neuro-2a neuronal cells were pretreated with PD98059 or U0126 (10 μM, for 30 min), followed by RSPO3 (50 ng/mL) treatment for 30 min, cells were then maintained under oxygen glucose deprivation (OGD) for 4 h and then re-oxygenation (“OGD/R”) for 48 h, cell viability and death were tested by CCK-8 (**C**) and medium LDH release (**D**) assays, respectively. “Mock” stands for the mock treatment (norm-oxygenated medium with glucose). Data were presented as mean ± standard deviation (SD, *n* = 5). *P < 0.001 *vs*. “Veh” treatment (**A**, **B**). ^#^*P* < 0.001 (**C**, **D**). Each experiment was repeated five times and similar results were obtained.

To study the signaling mechanism of RSPO3-induced neuroprotection, Neuro-2a cells were treated with RSPO3 (50 ng/mL) and levels of phosphorylated-Akt, phosphorylated-Erk1/2 and active (non-phosphorylated) β-Catenin were increased (Fig. 4A). Similarly, in primary murine cortical neurons RSPO3 treatment augmented Akt, Erk1/2 and active β-Catenin (Fig. 4B). Importantly, co-treatment with Erk inhibitors, including PD98059 and U0126, abolished RSPO3-induced neuroprotection against OGD/R in Neuro-2a cells (Fig. 4C, D). Conversely, the β-Catenin inhibitor FH535 and the Akt inhibitor LY294002 had no effect on RSPO3-induced neuroprotection against OGD/R in Neuro-2a cells (Fig. S2A, B). Notably, FH535 downregulated β-Catenin target gene *LEF1* expression in RSPO3-treated cells (Fig. S2C) and LY294002 blocked RSPO3-induced Akt phosphorylation (Fig. S2D). These results show that Erk activation is essential for RSPO3-induced neuroprotection against OGD/R.

### Gαi1 and Gαi3 are required for RSPO3-induced Erk activation and neuroprotection against OGD/R

**Fig. 5:**
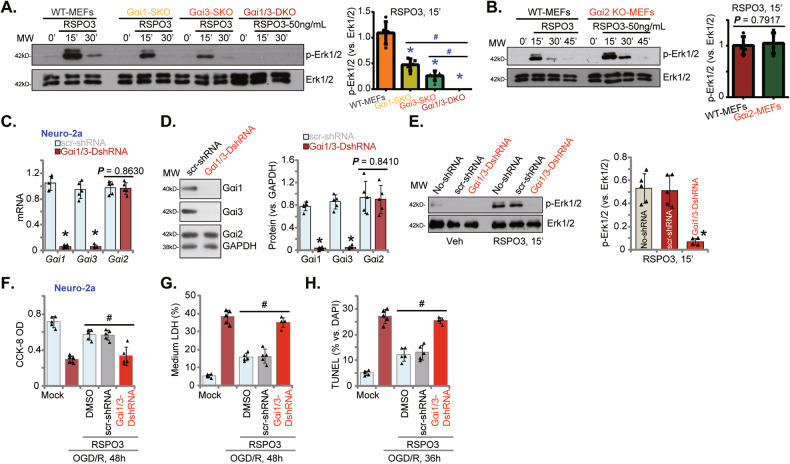
Gαi1 and Gαi3 are required for RSPO3-induced Erk activation and neuroprotection against OGD/R. Wild-type (WT), Gαi1/3 double knockout (DKO), Gαi1, Gαi2 or Gαi3 single knockout (SKO) mouse embryonic fibroblasts (MEFs) were treated with RSPO3 (at 50 ng/mL) and cultivated for indicated time periods, expression of listed proteins was shown (**A**, **B**). Neuro-2a cells, with the Gαi1 shRNA plus the Gαi3 shRNA (“Gαi1/3-DshRNA”), the scramble control shRNA (“scr-shRNA”), were established and expression of listed genes and proteins was shown (**C**, **D**); Cells were treated with RSPO3 (50 ng/mL) or vehicle control and cultured for 15 min, expression of listed proteins was tested (**E**). Gαi1/3-DshRNA or scr-shRNA Neuro-2a cells were pretreated with RSPO3 (50 ng/mL) for 30 min, cells were then maintained under oxygen glucose deprivation (OGD) for 4 h and then re-oxygenation (“OGD/R”) for the applied time periods, cell viability, death and apoptosis were tested by CCK-8 (**F**), medium LDH release (**G**) and nuclear TUNEL staining (**H**) assays, respectively. “Mock” stands for the mock treatment (norm-oxygenated medium with glucose). Data were presented as mean ± standard deviation (SD, *n* = 5). *P < 0.001 *vs*. “WT” MEFs/“scr-shRNA”. ^#^*P* < 0.001. Each experiment was repeated five times and similar results were obtained.

Our previous studies have established the critical roles of Gαi1 and Gαi3 (but not Gαi2) proteins in transducing Erk1/2 activation of multiple receptors [32, 34, 35, 42]. We therefore tested whether Gαi proteins are also important for RSPO3-induced Erk activation and neuroprotection. As shown, RSPO3-induced Erk1/2 phosphorylation was blocked in Gαi1 and Gαi3 double knockout (DKO) mouse embryonic fibroblasts (MEFs) (Fig. 5A). Gαi1 or Gαi3 single knockout (SKO) resulted in partial inhibition of Erk1/2 phosphorylation in RSPO3-treated MEFs (Fig. 5A). Gαi2 SKO in MEFs failed to significantly reduceRSPO3-induced Erk1/2 phosphorylation (Fig. 5B).

Neuro-2a cells were infected with Gαi1 shRNA lentivirus and Gαi3 shRNA lentivirus and stable cells selected (“Gαi1/3-DshRNA”). Control cells were stably transfected with scramble control shRNA lentivirus (“scr-shRNA”) (Fig. 5C, D). *Gαi1* and *Gαi3* mRNA and protein expression levels were robustly downregulated in Gαi1/3-DshRNA (Fig. 5C, D), leaving *Gαi2* mRNA and protein unchanged (Fig. 5C, D). Knockdown of Gαi1 and Gαi3 in Neuro-2a cells inhibited RSPO3-provoked Erk1/2 phosphorylation (Fig. 5E). These results support that Gαi1 and Gαi3 are required for RSPO3-induced Erk activation in neuronal cells. Remarkably, Gαi1/3 silencing reversed RSPO3-induced inhibition of OGD/R-caused viability reduction (Fig. 5F), cell death (Fig. 5G) and apoptosis (Fig. 5H).

### RSPO3 induces LGR4-Gab1-Gαi1/3 association, required for downstream Erk activation in neuronal cells

**Fig. 6:**
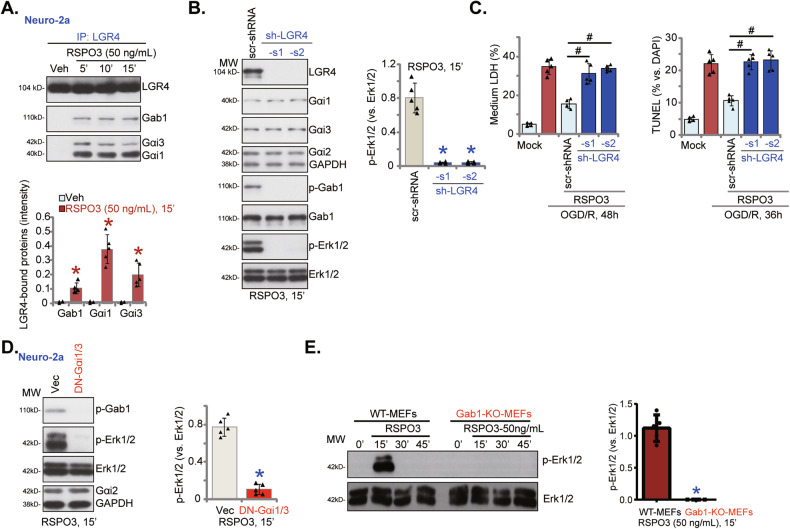
RSPO3 induces LGR4-Gab1-Gαi1/3 association, required for downstream Erk activation in neuronal cells. Neuro-2a cells were treated with RSPO3 (50 ng/mL) and cultivated for 5–15 min, LGR4 association with Gab1 and Gαi1/3 was examined by co-immunoprecipitation (“Co-IP”) assays (**A**); Neuro-2a cells, stably expressing the lentiviral LGR4 shRNA (sh-LGR4-s1 and sh-LGR4-s2, representing two different sequences) or the scramble control shRNA (“scr-shRNA”), were treated with RSPO3 (50 ng/mL) and cultured for 15 min, expression of listed proteins was tested (**B**); Alternatively, cells were pretreated with RSPO3 (50 ng/mL) for 30 min, cells were then maintained under oxygen glucose deprivation (OGD) for 4 h and then re-oxygenation (“OGD/R”) for the applied time periods, cell death and apoptosis were tested by medium LDH release and nuclear TUNEL staining assays, respectively (**C**). Neuro-2a cells, stably expressing the dominant negative (DN)-Gαi1 plus DN-Gαi3 (“DN-Gαi1/3”) or the empty vector (“Vec”), were treated with RSPO3 (50 ng/mL) and cultured for 15 min, expression of listed proteins was shown (**D**). Wild-type (WT) and Gab1 knockout (KO) mouse embryonic fibroblasts (MEFs) were treated with RSPO3 (50 ng/mL) and cultivated for 15–45 min, expression of listed proteins was shown (**E**). Data were presented as mean ± standard deviation (SD, *n* = 5). *P < 0.001 *vs*. “Veh”/“scr-shRNA”/“Vec”/“WT MEFs”. ^#^*P* < 0.001 (**C**). Each experiment was repeated five times and similar results were obtained.

As our recent study reported that RSPO3 induced Gαi1/3 association with LGR4 and Gab1 in endothelial cells, mediating downstream Akt-mTOR activation to promote angiogenesis [31], we explored whether RSPO3-induced LGR4-Gab1-Gαi1/3 association is important for Erk activation. Co-immunoprecipitation (Co-IP) assay results in Neuro-2a cells revealed that RSPO3 (50 ng/mL) promotes LGR4-Gab1-Gαi1/3 association (Fig. 6A). Silencing LGR4, using stable Neuro-2a cells expressing LGR4 shRNAs [31], robustly decreased LGR4 protein expression and inhibited RSPO3 (50 ng/mL)-provoked Gab1 and Erk1/2 phosphorylation in Neuro-2a cells, without affecting Gαi1/2/3, Gab1 and Erk1/2 protein expression (Fig. 6B). In Neuro-2a cells, RSPO3-mediated inhibition of OGD/R-induced cell death and apoptosis was abolished by shRNA-induced silencing of LGR4 (Fig. 6C).

To further support that LGR4-Gab1-Gαi1/3 association is required for RSPO3-induced Erk1/2 activation, dominant negative (“DN”) mutants of Gαi1 and Gαi3 (“DN-Gαi1/3”) were transduced into Neuro-2a cells. These Gαi1/3 mutants replace the conserved Gly (G) residue with Thr (T) in the G3 box preventing Gαi1/3 association with adaptor/associated proteins [37, 62]. As shown, DN-Gαi1/3 inhibited Gab1 and Erk1/2 activation in response to RSPO3 in Neuro-2a cells (Fig. 6D). RSPO3-induced Erk1/2 phosphorylation was abolished in Gab1 KO MEFs (Fig. 6E).

### Endothelial knockdown of RSPO3 enhances MCAO-caused cerebral ischemic injury

**Fig. 7:**
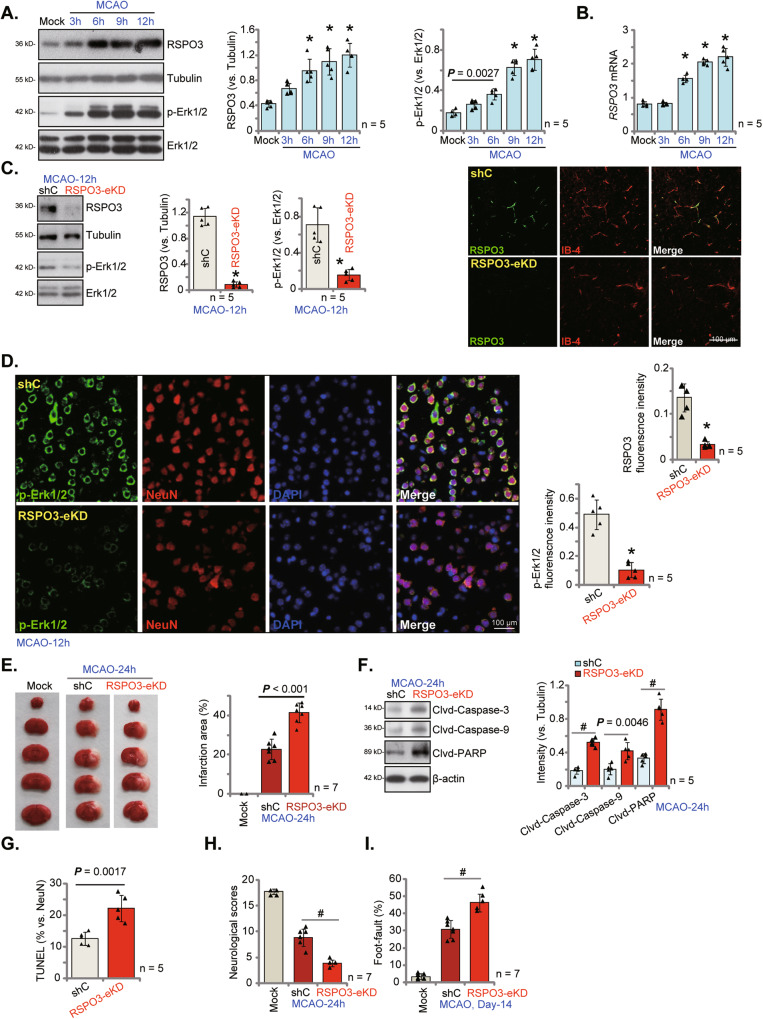
Endothelial knockdown of RSPO3 intensifies MCAO-caused cerebral ischemic injury. C57BL/6 J mice were subject to MCAO procedure for applied time periods, and the ischemic penumbra brain regions were isolated. Expression of listed genes and proteins in the brain tissues were tested (**A**, **B**). RSPO3 shRNA-expressing adenovirus (RSPO3 shRNA-TIE1-AAV5, “RSPO3-eKD”) or the scramble control shRNA-expressing adenovirus (TIE1-AAV5, “shC”) were injected to the lateral ventricle of the mice; After 20 days, the mice were subject to MCAO procedure, and the ischemic penumbra brain regions were isolated and expression of listed proteins was tested (**C**, **F**). The ischemic penumbra brain slides were subject to designated fluorescence staining and representative images were shown (**C**, the right panel, **D**). TTC staining was employed to stain the ischemic region and results were quantified (**E**). The brain slides were also subjected to TUNEL/NeuN staining and TUNEL-positive nuclei ratio was recorded (**G**). Other mice were subject to the behavior tests, the neurological scores were recorded (**H**, at 24 h) and foot-fault tests (**I**, at Day-14) were performed. Data were presented as mean ± standard deviation (SD). *P < 0.001 *vs*. “Mock”/“shC” group. ^#^*P* < 0.001. In each group there were five/seven mice (*n* = 5/7). Scale bar = 100 μm.

We next examined whether the expression of RSPO3 is changed in mouse brain tissue after cerebral ischemia/reperfusion. Ischemic penumbra brain tissues were collected 3 h, 6 h, 9 h and 12 h following MCAO. As shown, RSPO3 protein levels gradually increased in the ischemic penumbra brain tissues of MCAO mice (Fig. 7A). Moreover, *RSPO3* mRNA in brain tissues was significantly upregulated 6–12 h after MCAO (Fig. 7B). Notably, RSPO3 protein was high in primary murine brain endothelial cells (VACM-1-positve, “Endothelial cells”) (Fig. S3A, B). In contrast, RSPO3 expression was extremely low in NeuN-positive primary murine cortical neurons and GFAP-positive primary murine astrocytes (Fig. S3A), and null in primary murine microglia (Iba-1-positve staining, Fig. S3A). In a time-dependent manner, OGD/R stimulation increased mRNA (Fig. S3C) and protein (Fig. S3D) expression of RSPO3 in primary murine brain endothelial cells. Next, the medium samples, free of debris and cells, were collected and were centrifuged. The supernatant containing secreted proteins was thereafter collected and was tested by Western blotting assays. Results show that production of RSPO3 in the medium was significantly increased in OGD/R-stimulated primary murine endothelial cells (Fig. S3E).

To demonstrate the neuroprotective activity of RSPO3 in vivo, RSPO3 was specifically knocked down in endothelial cells using an shRNA-expressing adenovirus (AAV5 construct, containing TIE1 promoter region) injected into the lateral ventricle: “RSPO3-eKD”. The control mice received lateral ventricle injection of scramble control shRNA adenovirus (“shC”, AAV5-TIE1 construct). After 20 days, the MCAO procedure was applied to both RSPO3-eKD and shC mice. Twelve hours later, MCAO-induced RSPO3 expression and Erk activation were largely inhibited in the RSPO3-eKD mice’s ischemic penumbra brain tissue (Fig. 7C). RSPO3 and IB-4 (the endothelial cell marker [42, 52]) double fluorescence staining results in the mouse brain sections confirmed in vivo endothelial knockout of RSPO3 in RSPO3-eKD mice (Fig. 7C, the right panel). RSPO3 green fluorescence signaling was primarily localized in IB-4-positive endothelial cells in shC control mice (Fig. 7C, the right panel). Mouse brain immunofluorescence staining further confirmed Erk inhibition in neurons (NeuN-positive staining) of MCAO-treated RSPO3-eKD mice (Fig. 7D).

In RSPO3-eKD mice, MCAO cerebral ischemic injury significantly enlarged the infarct area (TTC staining) (Fig. 7E), and RSPO3 endothelial silencing augmented MCAO-induced neuronal apoptosis. Cleaved-caspase-3/caspase -9/PARP levels were increased in the ischemic penumbra brain tissues of RSPO3-eKD mice (Fig. 7F). TUNEL/NeuN fluorescence staining showed that RSPO3-eKD augmented MCAO-induced apoptosis and increased TUNEL-positive neurons in ischemic penumbra brain tissues of MCAO mice (results quantified in Fig. 7G). After MCAO, the neurological scores were much worse in RSPO3-eKD mice (Fig. 7H). The foot-fault ratio, tested 14 days after MCAO, was also significantly higher in RSPO3-eKD mice (Fig. 7I).

To study the potential neuroprotective effect of endothelial cell-derived RSPO3 in vitro, treatment of primary murine cortical neurons with the conditioned medium of OGD/R-stimulated primary murine brain endothelial cells (“ECs”) inhibited the OGD/R-induced viability reduction (Fig. S3F) and cell death (Fig. S3G). The neuroprotective effect of the conditioned medium of murine brain endothelial cells was largely compromised after silencing RSPO3 (through lv-RSPO3-eKD lentivirus) (Fig. S3F, G).

### Endothelial conditional knockout of RSPO3 exacerbates MCAO-induced cerebral ischemic injury

**Fig. 8:**
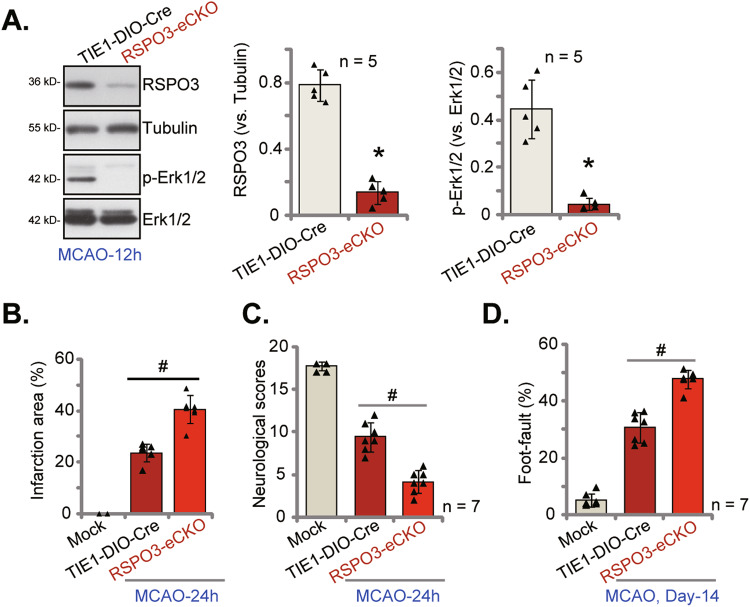
Endothelial conditional knockout of RSPO3 exacerbates MCAO-induced cerebral ischemic injury. AAV5-FLEX-CRISPR/Cas9-RSPO3-KO was injected into the lateral ventricle of the TIE1-DIO-Cre C57 mice (4-week old, male). After 20 days, RSPO3 endothelial conditional knockout (RSPO3-eCKO) mice were established. RSPO3-eCKO mice and TIE1-DIO-Cre control mice were subject to MCAO procedure. After indicated time periods, the ischemic penumbra brain regions were isolated and expression of listed proteins was tested (**A**). TTC staining was employed to stain the ischemic region and results were quantified (**B**). Other mice were subject to the behavior tests, the neurological scores were recorded (**C**, at 24 h) and foot-fault tests (**D**, at Day-14) were carried out. Data were presented as mean ± standard deviation (SD). *P < 0.001 *vs*. “TIE1-DIO-Cre” group (**A**). ^#^*P* < 0.001 (**B**–**D**). In each group there were five/seven mice (*n* = 5/7).

To further support the role of endothelial cell-derived RSPO3 against cerebral ischemic injury, the AAV5-FLEX-CRISPR/Cas9-RSPO3-KO was injected into the lateral ventricle of the TIE1-DIO-Cre C57 mice [52], thereby generating RSPO3 endothelial conditional knockout (RSPO3-eCKO) mice. After 20 days, the MCAO procedure was applied to RSPO3-eCKO mice and TIE1-DIO-Cre control mice. The ischemic penumbra brain tissue was collected 12 h after MCAO, and tissue lysates tested. As shown, RSPO3 protein expression and Erk1/2 phosphorylation were substantially decreased in brain tissues of RSPO3-eCKO mice (Fig. 8A). RSPO3-eCKO dramatically intensified MCAO-induced cerebral ischemic injury and the infarct area (TTC staining) was substantially enlarged (results quantified in Fig. 8B). When compared to control TIE1-DIO-Cre mice, the neurological scores were much worse in RSPO3-eCKO mice with MCAO (Fig. 8C). Moreover, the foot-fault ratio was significantly higher in RSPO3-eCKO MCAO mice (Fig. 8D). Therefore, RSPO3-eCKO exacerbated MCAO-induced cerebral ischemic injury.

### Endothelial RSPO3 overexpression ameliorates MCAO-induced cerebral ischemic injury

**Fig. 9:**
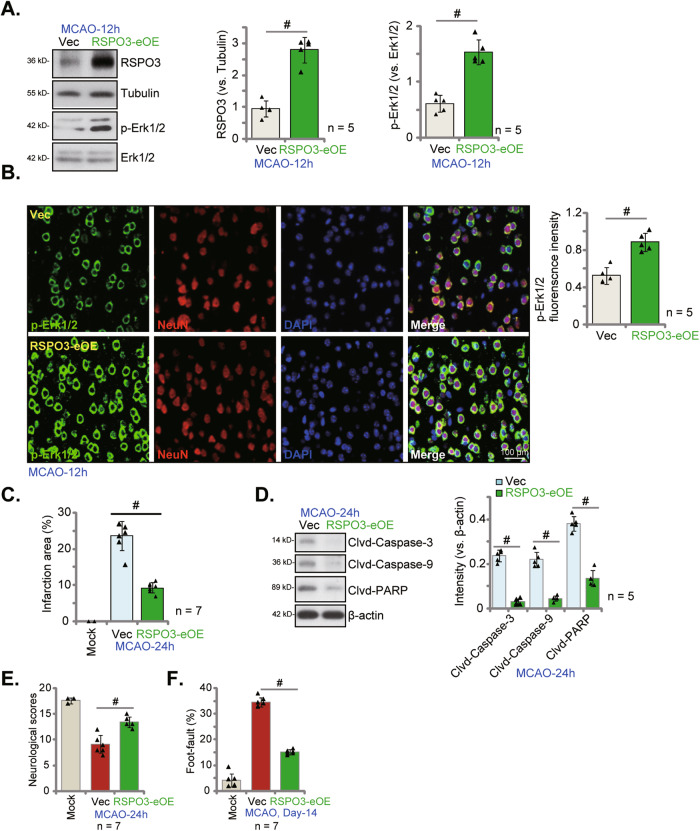
Endothelial RSPO3 overexpression ameliorates MCAO-induced cerebral ischemic injury. The RSPO3-overexpressing adenovirus with TIE1 promoter region (*RSPO3* cDNA-TIE1-AAV5) was injected to mouse lateral ventricle (“RSPO3-nOE” group); The control group mice were injected with the TIE1-AAV5 empty vector adenovirus (“Vec”); After 20 days, MCAO was applied to the mice. After indicated time periods, the ischemic penumbra brain regions were isolated and expression of listed proteins in the brain tissues was measured (**A**, **D**). The ischemic penumbra brain slides were subject to designated fluorescence staining and representative images were shown (**B**). The ischemic region was stained by TTC and results were quantified (**C**). Mice were also subject to the behavior tests, the neurological scores were recorded (**E**, at 24 h) and foot-fault tests (**F**, at Day-14) were performed. Data were presented as mean ± standard deviation (SD). ^#^*P* < 0.001. In each group there were five/seven mice (*n* = 5/7). Scale bar = 100 μm.

Based on the above RSPO3-eKD/-eCKO results, we examined whether endothelial overexpression of RSPO3 could potentially inhibit cerebral ischemic injury in mice. To test this, RSPO3-expressing adenovirus (AAV5, with TIE1 promoter region) was injected into the lateral ventricle, with the aim of overexpressing endothelial RSPO3 (“RSPO3-eOE”). Control mice were injected with empty vector AAV5 (“Vec”). Twenty days later, the MCAO procedure was performed, and the ischemic penumbra brain tissues isolated after 12 h. RSPO3 protein expression was robustly elevated in ischemic penumbra brain regions in RSPO3-eOE mice (Fig. 9A), and Erk activation augmented (Fig. 9A). The p-Erk fluorescence staining in ischemic penumbra brain tissue sections demonstrated that Erk activation was significantly increased in RSPO3-eOE MACO mice (Fig. 9B).

RSPO3-eOE significantly inhibited MCAO-induced cerebral ischemic injury in mice. MCAO induced infarct area was reduced in the RSPO3-eOE mice (Fig. 9C), and cleavage of apoptosis proteins inhibited (Fig. 9D), supporting that RSPO3-eOE inhibited MCAO-induced apoptosis. The neurological scores were improved in RSPO3-eOEMCAO mice (Fig. 9E), and the foot-fault ratio significantly reduced (Fig. 9F). Thus, RSPO3-eOE increases Erk activation and inhibits MCAO-induced cerebral ischemic injury in mice.

## Discussion

The results of the present study demonstrate the neuroprotective function of RSPO3. In Neuro-2a cells and primary murine cortical neurons, RSPO3 pretreatment largely ameliorated OGD/R-induced oxidative injury, neuronal cell death and apoptosis. In vivo, RSPO3 expression was increased in the ischemic penumbra brain tissues of MCAO mice. Endothelial knockdown or endothelial conditional knockout of RSPO3 intensified MCAO-induced cerebral ischemic injury. Endothelial RSPO3 overexpression ameliorated cerebral ischemic injury, demonstrating that endothelial cell-derived RSPO3 protects neuronal cells from ischemia/reperfusion injury.

Recent studies have reported that activation of the Erk signaling cascade by a number of different agents can exert significant neuroprotection against ischemic injury in vitro and in vivo. Liu et al. reported that heparin activation of the Erk-CREB-PTN-syndecan-3 cascade attenuated ischemia/reperfusion neuronal injury [63]. Zhao et al. demonstrated that cytosine inhibits cerebral ischemia/reperfusion injury in mice by activating the NR2B-ERK-CREB cascade [64]. Quercetin activates both Erk and Akt signaling to protect against neuronal ischemia/reperfusion injury [65]. Feng et al. reported that a novel diarylacyl hydrazone derivative A11 ameliorated ischemic injury via activation of Erk [66].

The role of RSPO3 on Erk signaling is inconsistent between different studies, which could be dependent on different cell types. Chen et al. reported that RSPO3 promoted JEG-3 cell growth by activating Erk and Akt signaling cascades [67]. Similarly, Gu et al. found that loss of RSPO3 resulted in decreased Erk phosphorylation in prostate cancer cells [68]. However, RSPO3 silencing was found to enhance Erk signaling in human adipose-derived stem cells [69]. We show that RSPO3 activates the Akt, Erk and β-Catenin cascade in neuronal cells and murine neurons. Significantly, Erk inhibitors reversed RSPO3-induced neuroprotection against OGD/R. Endothelial knockdown of RSPO3 inhibited Erk activation in the ischemic penumbra brain tissues, while endothelial RSPO3 overexpression enhanced it. These results support that RSPO3 activates Erk signaling to protect neuronal cells from ischemia/reperfusion injuries.

Our group has established the essential roles of Gαi1/3 proteins in transducing the signaling of multiple receptors. Gαi1/3 associated with epidermal growth factor (EGF)-activated EGFR is required for downstream Akt-mTOR activation [62]. Following brain-derived neurotrophic factor (BDNF) stimulation, Gαi1/3 associates with TrkB to mediate downstream Akt-mTOR cascade activation [34]. Interestingly, Gαi1/3, by forming a complex with CD14 and Gab1, is indispensable for mediating lipopolysaccharide (LPS)-induced signaling [70]. With interleukin 4 (IL-4) stimulation Gαi1/3 associated with the intracellular domain of IL-4Rα, promoting IL-4Rαtraffic and downstream Akt activation in macrophages [36]. A very recent study from our group found that Gαi1/3 are also important for RSPO3-induced Akt-mTOR cascade activation, thereby promoting angiogenesis [31]. Gαi1/3 associated with RSPO3-stimulated LGR4 and Gab1 to transduce downstream Akt-mTOR activation [31].

Recent studies have proposed a pivotal role of Gαi1/3 in mediating Erk cascade activation. Li et al. have shown that in MEFs and endothelial cells, Netrin-1 treatment induced Gαi1/3 association with its receptor CD146, leading to CD146 internalization, Gab1 recruitment and downstream Erk activation [32]. Shan et al. discovered that Gαi1/3 can associate with SCF (stem cell factor)-stimulated receptor c-Kit in endothelial cells, causing c-Kit endocytosis and recruitment of adaptor proteins, thereby promoting downstream Erk activation [42]. Sun et al. reported that Gαi1/3 was in the VEGFR2 (the VEGF receptor) endocytosis complex, required for VEGF-induced VEGFR2 endocytosis and downstream Erk activation [33]. Gαi1/3 silencing, KO or DN mutation suppressed VEGF-induced Erk activation [33]. Wang et al. found that neuroligin-3 (NLGN3)-induced Erk activation also required Gαi1 and Gαi3 in glioma cells [35].

**Fig. 10:**
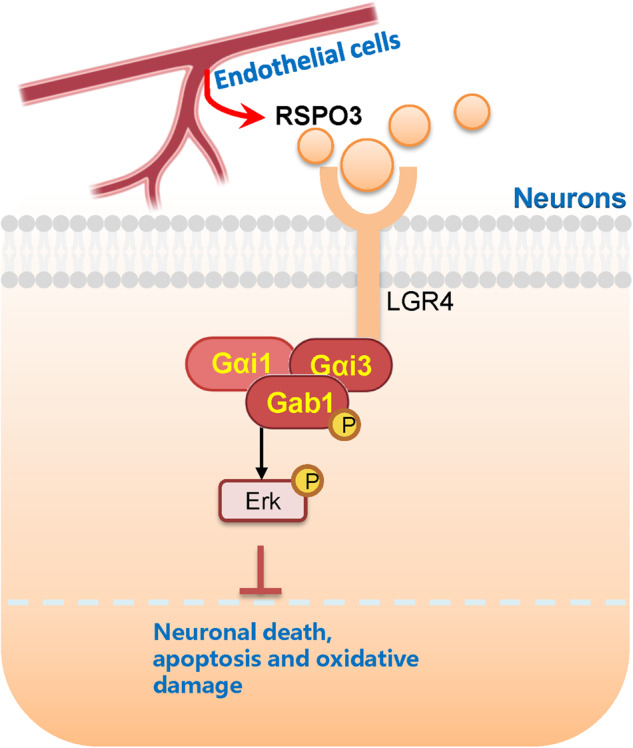
The proposed signaling cascade of the present study.

Here we further show that Gαi1/3 proteins are vital for Erk activation. Importantly, RSPO3-induced Erk activation in neuronal cells was suppressed by Gαi1/3 silencing, but augmented following ectopic Gαi1/3 overexpression. RSPO3 induced LGR4-Gab1-Gαi1/3 association was required for downstream Erk activation in neuronal cells. LGR4-Gab1 depletion largely inhibited RSPO3-induced Erk activation. An early study from our group discovered that knockdown of Gαi1 and Gαi3 decreased the number of dendrites and dendritic spines in hippocampal neurons [34]. Here,Gαi1/3 silencing reversed RSPO3-induced neuroprotection against OGD/R. Together, these results demonstrate that RSPO3 activates LRG4-Gαi1/3-Gab1-Erk signaling cascade, offering significant neuroprotection against cerebral ischemic injury (Fig. 10).

Interestingly, despite the high sequence homology, Gαi1 and Gαi3, but not Gαi2, are key signaling proteins for RSPO3-induced Erk activation and neuronal protection. RSPO3 induces LGR4-Gab1-Gαi1/3 association in neuronal cells, whereas Gαi2 was not in the signaling complex. Xu et al. showed that Gαi2 depletion failed to affect RSPO3-induced Akt-mTOR activation in endothelial cells [31]. Early studies have also demonstrated that Gαi2 was not required for transducing signaling for RTKs and several non-RTK receptors [32–34, 42, 62, 70].

## Conclusion

RSPO3 activates Gαi1/3-Erk signaling to protect neuronal cells from ischemia/reperfusion injury. RSPO3 is a secretory protein. Our in vitro studies show that pretreatment with RSPO3 ameliorated OGD/R-induced oxidative injury and cell death in primary murine cortical neurons. In vivo, endothelial RSPO3 overexpression ameliorated MCAO-induced cerebral ischemic injury in mice. Therefore, targeting RSPO3 signaling pathway may have important therapeutic value for the treatment of ischemic stroke and other neuronal ischemia-reperfusion disorders.

### Supplementary information

Supplementary Figures

Original Data File

aj-checklist FORM

Author contribution

## Data Availability

The data are included in the article.
