# Supplementary material for: Endothelial cell-derived RSPO3 activates Gαi1/3-Erk signaling and protects neurons from ischemia/reperfusion injury
Source: Cell Death Dis. 2023 Oct 7;14(10):654. doi: 10.1038/s41419-023-06176-2 (PMC10560285; doi:10.1038/s41419-023-06176-2)

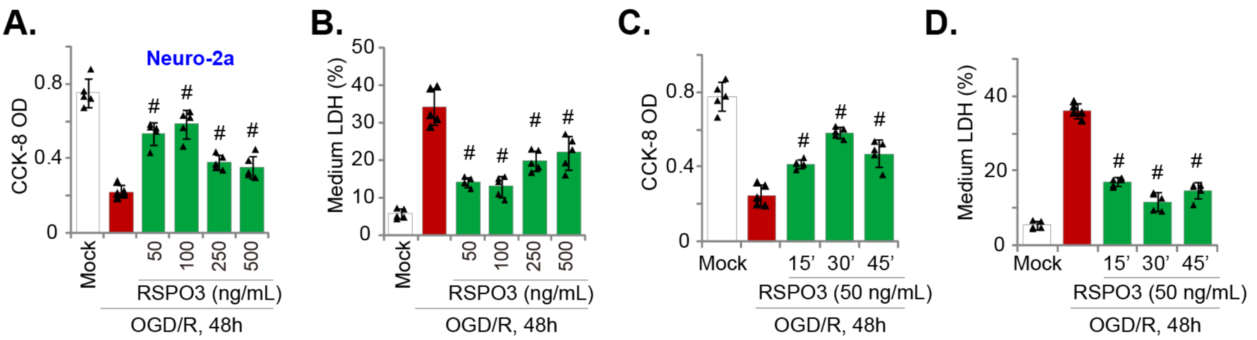

Figure S1. Neuro-2a neuronal cells were pretreated with RSPO3 (at described concentrations) for 30 min, followed by oxygen glucose deprivation (OGD) for 4h and then re-oxygenation (OGD/R), viability and cell death were respectively tested by CCK-8 (**A**) and medium LDH releasing (**B**) assays after 48h. Neuro-2a neuronal cells were pretreated with RSPO3 (at 50 ng/mL) for indicated time periods (15’-60’), followed by OGD/R stimulation, cell viability (**C**) and death (**D**) were measured similarly after 48h. “Mock” stands for the mock treatment (norm-oxygenated medium with glucose). Data were presented as mean ± standard deviation (SD, n=5). $^{\#}$***P*** <0.001 *vs.* OGD/R treatment. Each experiment was repeated five times and similar results were obtained.

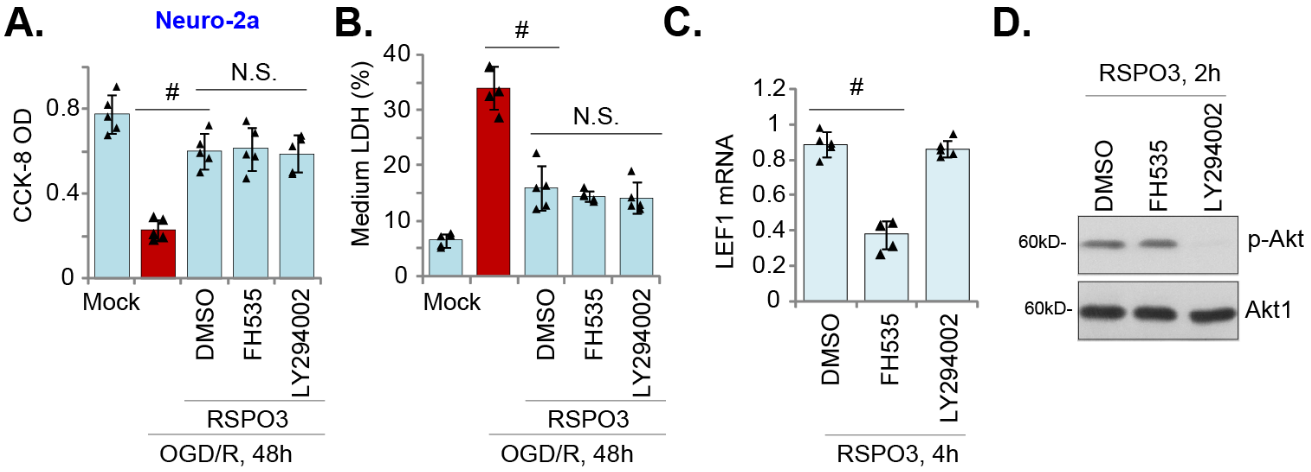

Figure S2. Neuro-2a neuronal cells were pretreated with FH535 (10 μM, for 30 min) or LY294002 (5 μM, for 30 min), followed by RSPO3 (50 ng/mL) treatment for 30 min, cells were then maintained under oxygen glucose deprivation (OGD) for 4h and then re-oxygenation ("OGD/R") for 48h, cell viability and death were tested by CCK-8 (**A**) and medium LDH release (**B**) assays, respectively. Neuro-2a neuronal cells were pretreated with FH535 (10 μM, for 30 min) or LY294002 (5 μM, for 30 min), followed by RSPO3 (50 ng/mL) treatment for 2-4h, expression of *LEF1* mRNA (Wnt/β-catenin target gene) was tested (**C**); Akt phosphorylation and expression were tested as well (**D**). Data were presented as mean ± standard deviation (SD, n=5). $^{\#}$ ***P*** <0.001. "N.S." stands for non-statistical different (*P* > 0.05).

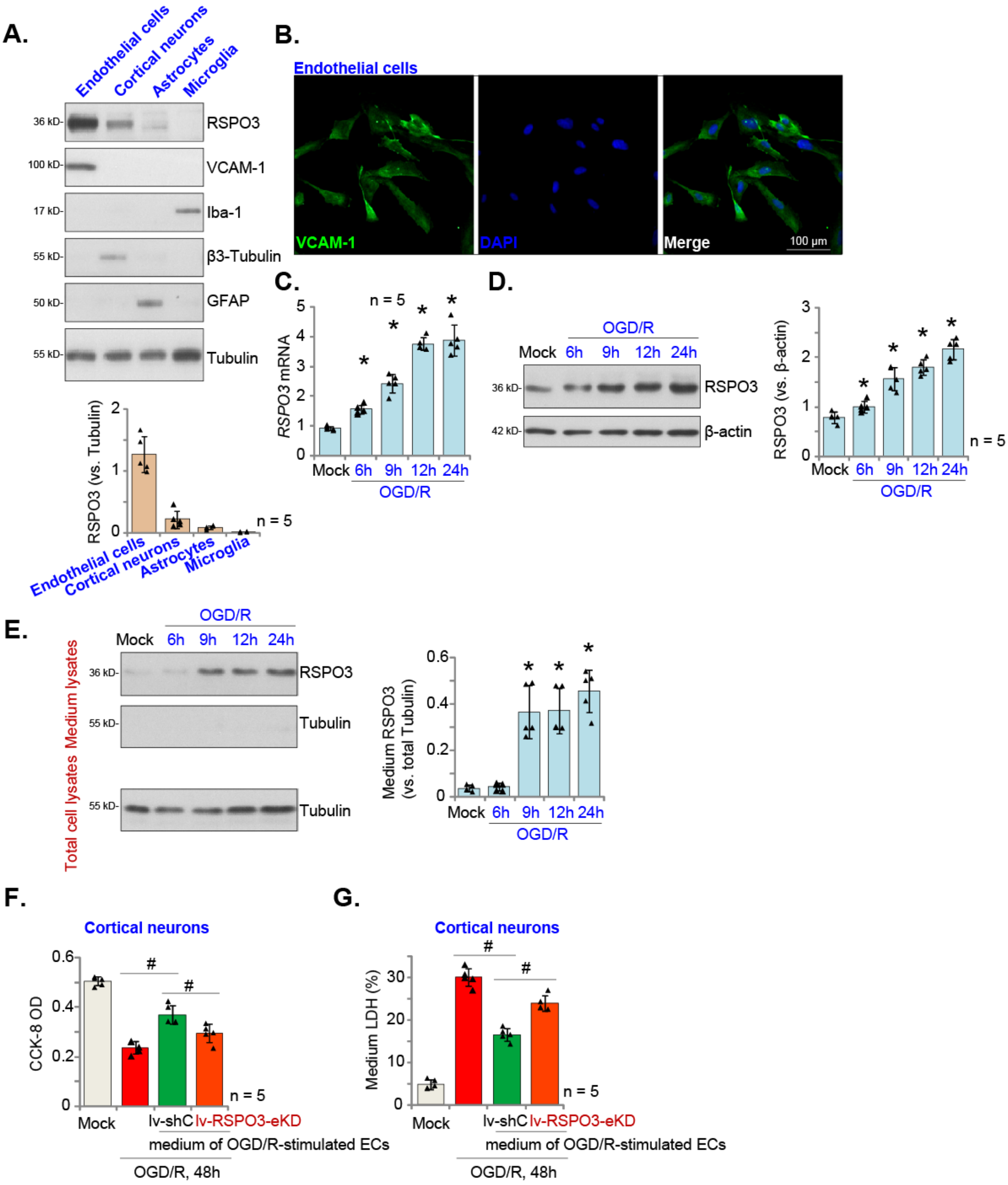

Figure S3. Expression of RSPO3 and listed proteins in primary murine brain endothelial cells ("Endothelial cells") as well as in primary murine cortical neurons, astrocytes and microglia was shown (**A**). The purity of primary murine brain endothelial cells ("Endothelial cells") was verified by VCAM-1 fluorescence staining (**B**). The primary murine brain endothelial cells ("Endothelial cells") were subject to oxygen glucose deprivation (OGD) for 4h and then re-oxygenation (OGD/R) for applied time periods, *RSPO3* mRNA (**C**) and protein (**D**) expression in total cell lysates was shown; The medium proteins were also collected and RSPO3

production was tested by Western blotting (**E**). The primary murine brain endothelial cells ("Endothelial cells") expressing lv-RSPO3-eKD or lv-shC were subject to OGD/R stimulation for 12h, medium was then collected and added to the primary cortical neurons (at 1 : 1 ratio). Neurons were then subject to OGD/R stimulation and cultivated for additional 48h, cell viability and death were tested by CCK-8 (**F**) and medium LDH releasing (**G**) assays, respectively. Data were presented as mean ± standard deviation (SD). "Mock" stands for the mock treatment (norm-oxygenated medium with glucose). * ***P*** <0.001 vs. "Mock" (**C**-**E**). # ***P*** <0.001 (**F** and **G**). Each experiment was repeated five times and similar results were obtained.

Figure S4: The un-cropped blotting images.

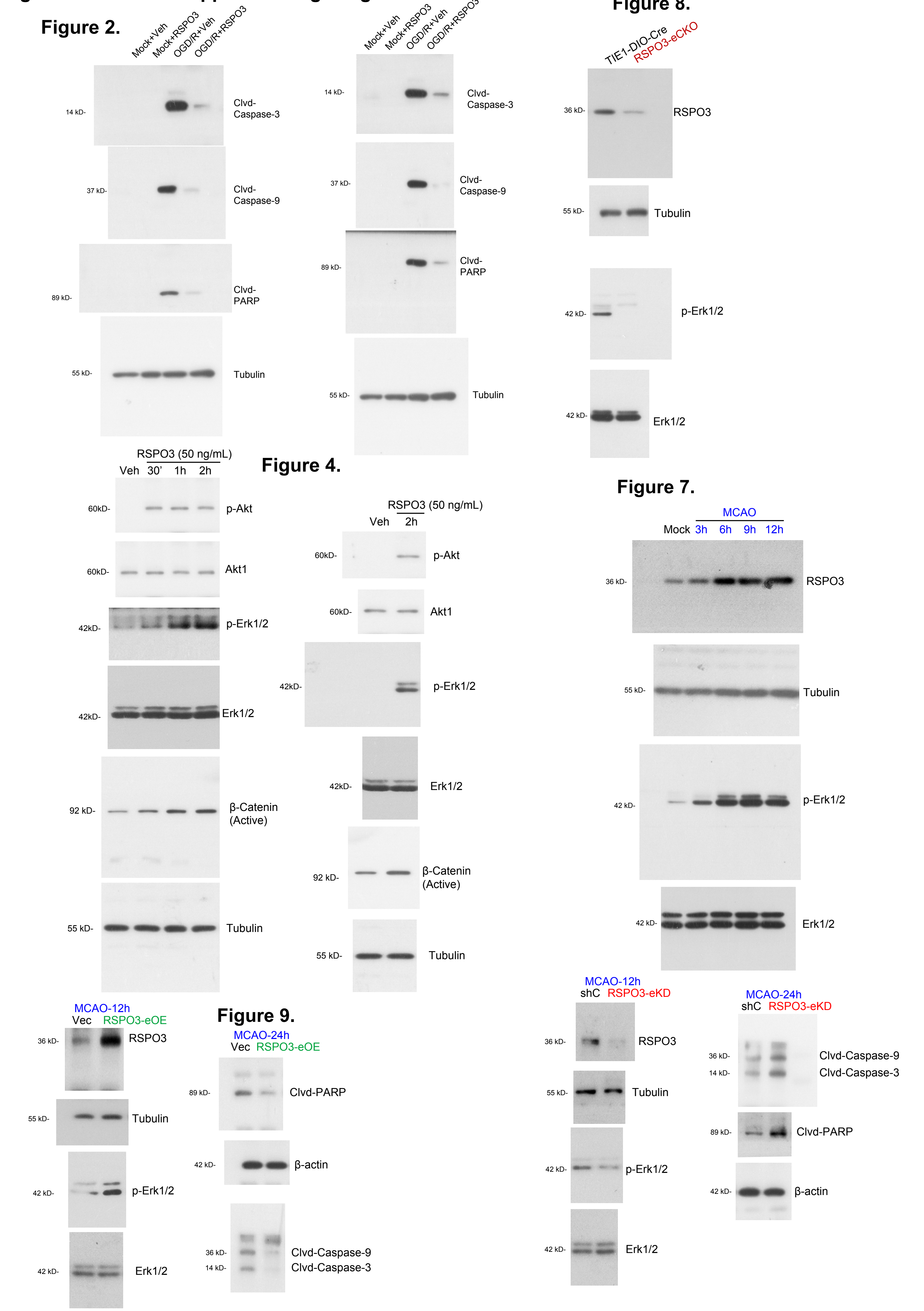

Figure 5.

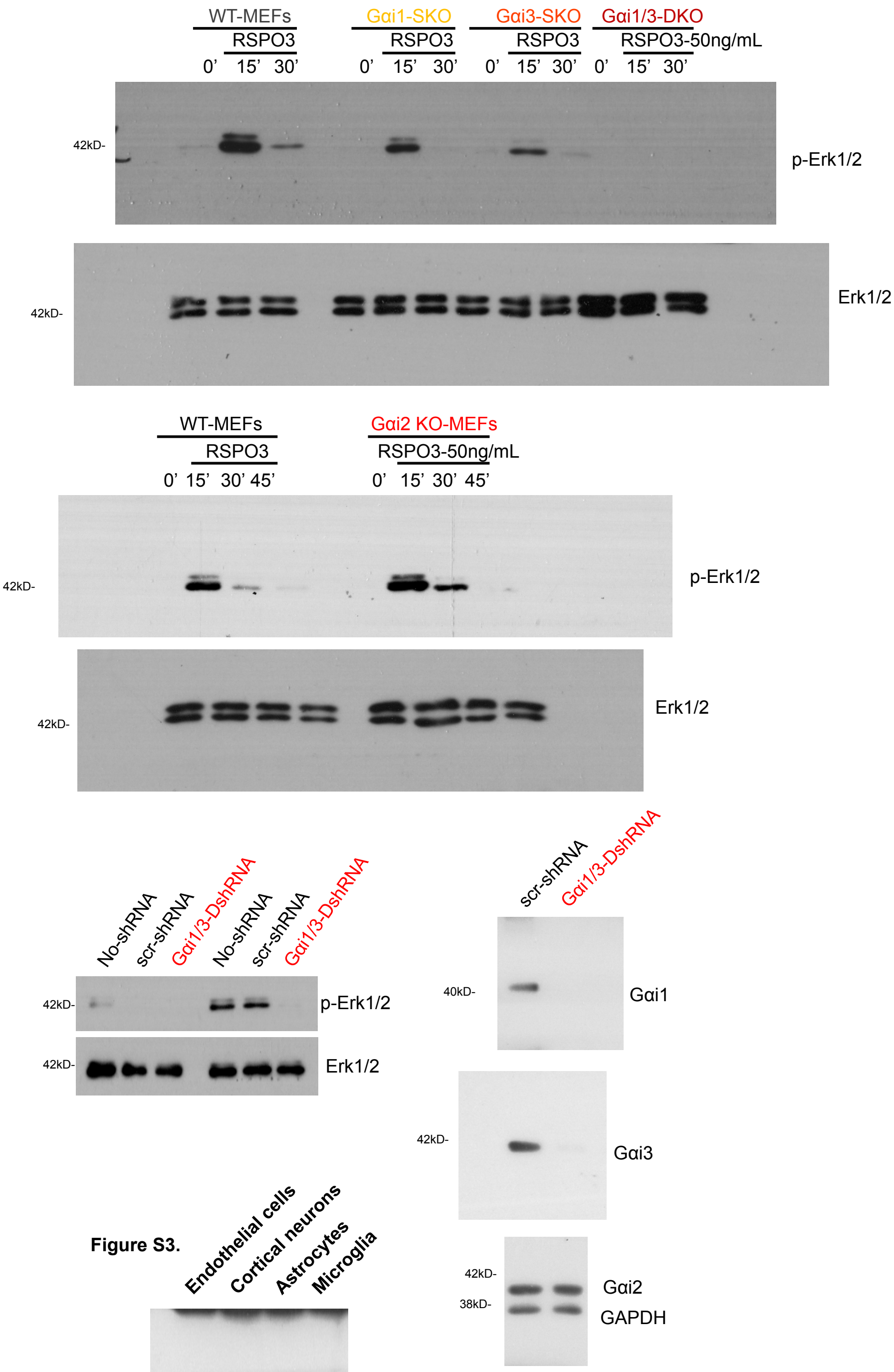

Figure 6

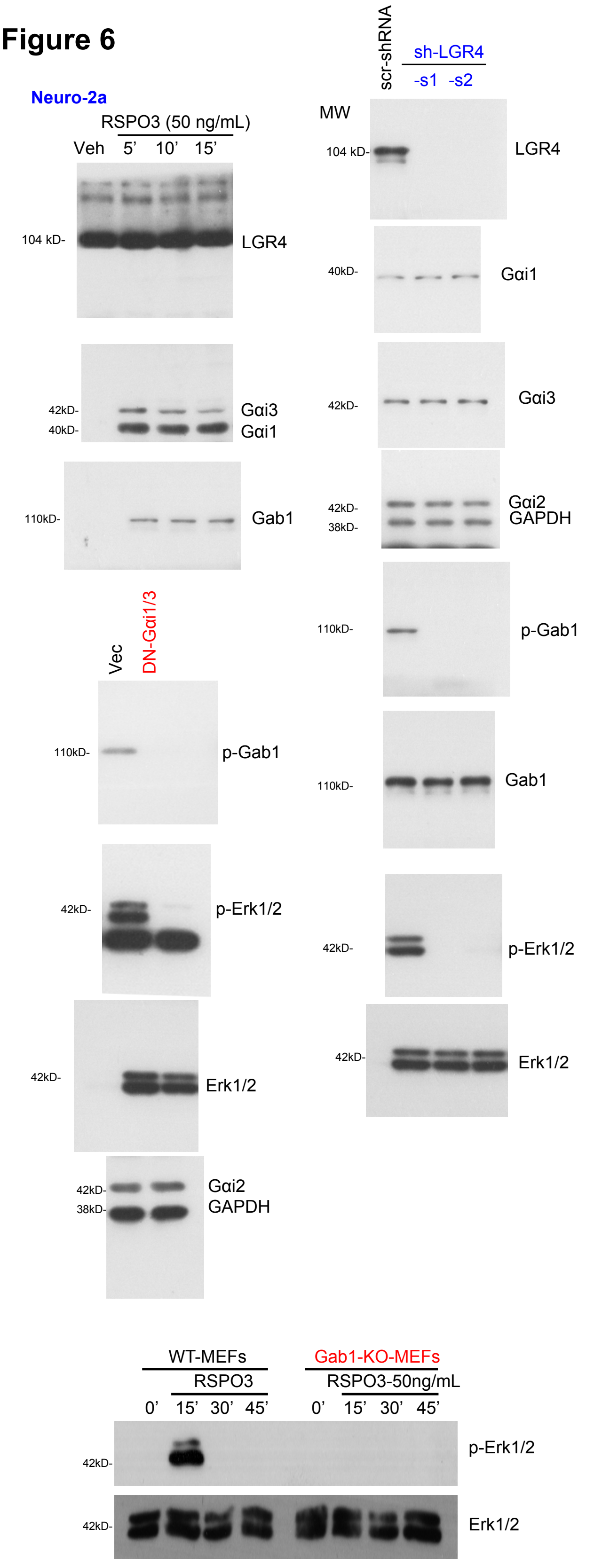

Figure S3.

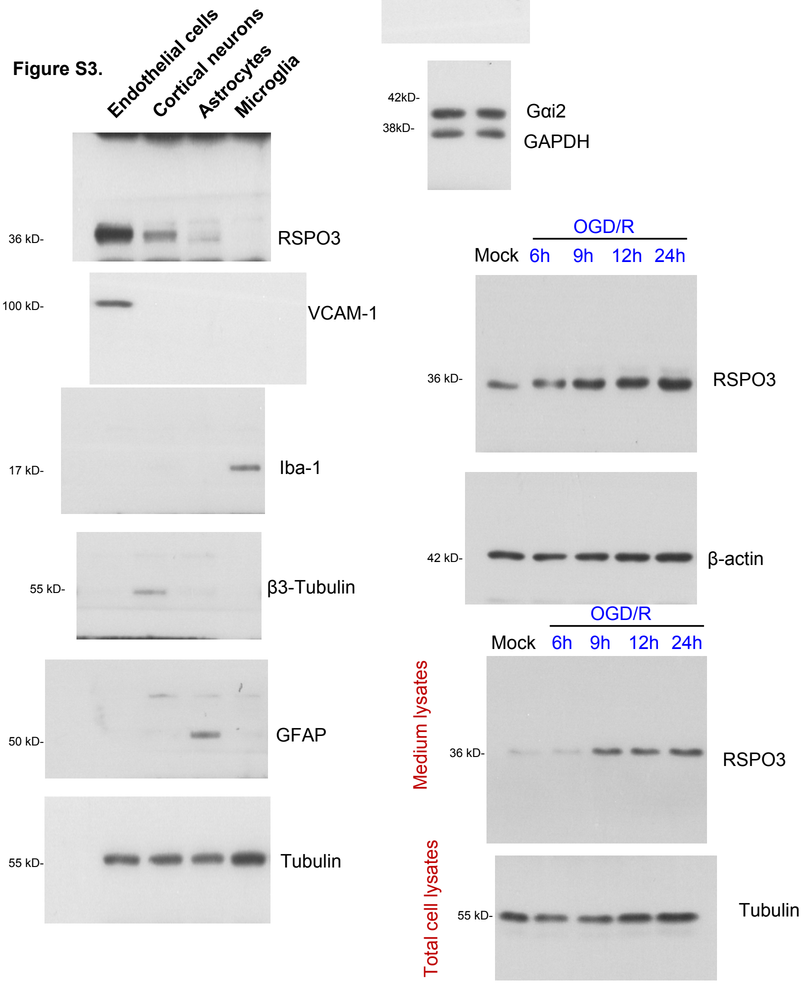

Figure S2.

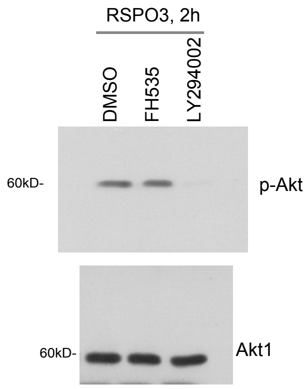

Supplement: Supplementary file 1 — Supplementary Figures [file 41419_2023_6176_MOESM1_ESM.pdf]
