# Supplementary material for: Endothelial cell-derived RSPO3 activates Gαi1/3-Erk signaling and protects neurons from ischemia/reperfusion injury
Source: Cell Death Dis. 2023 Oct 7;14(10):654. doi: 10.1038/s41419-023-06176-2 (PMC10560285; doi:10.1038/s41419-023-06176-2)

Figure S4: The un-cropped blotting images.

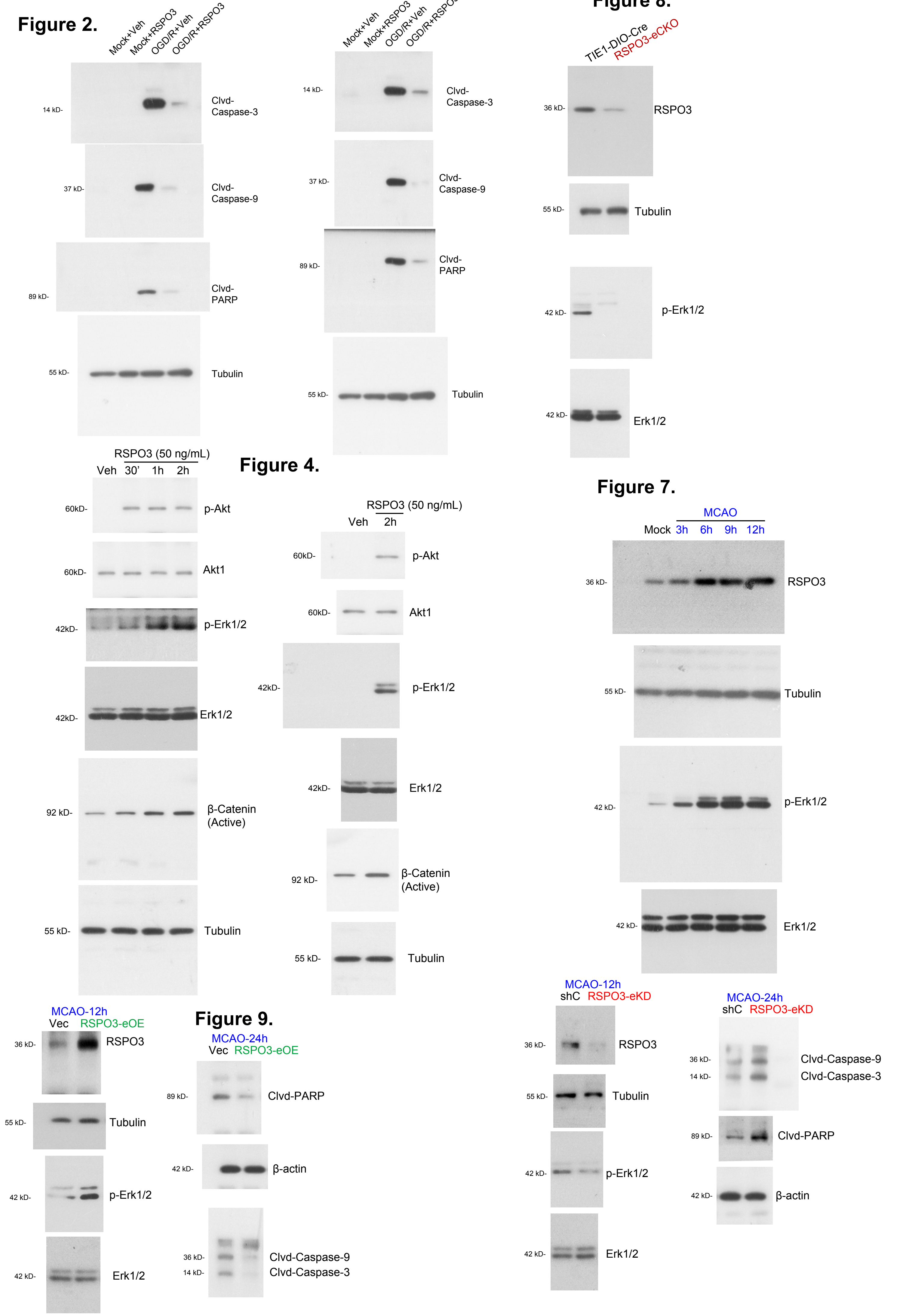

Figure 5.

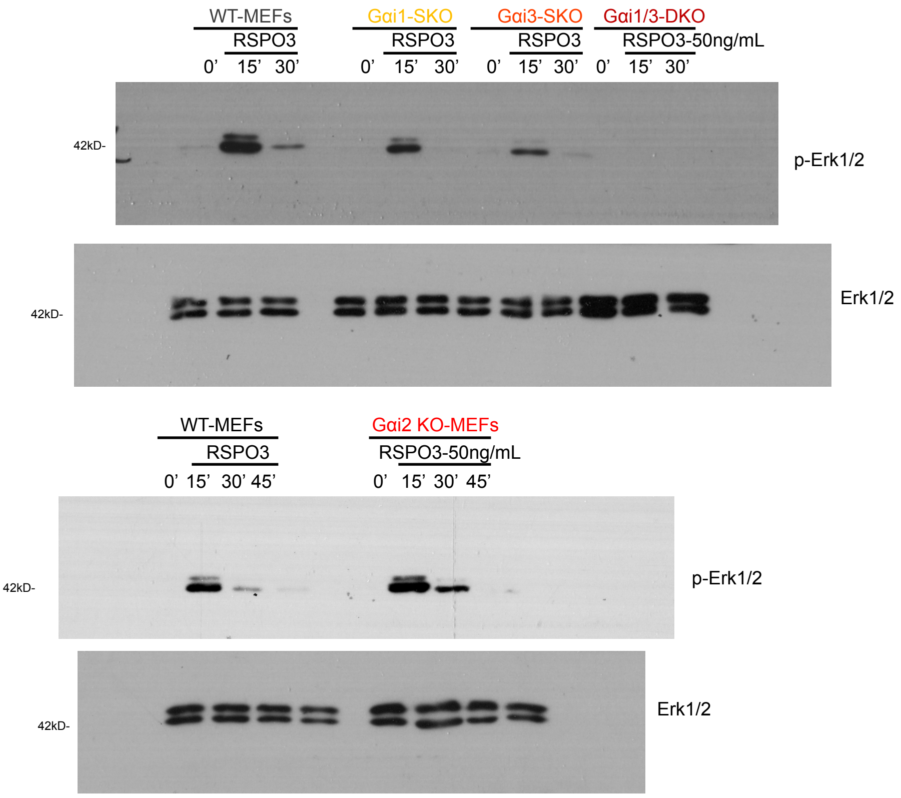

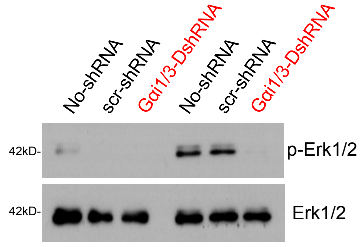

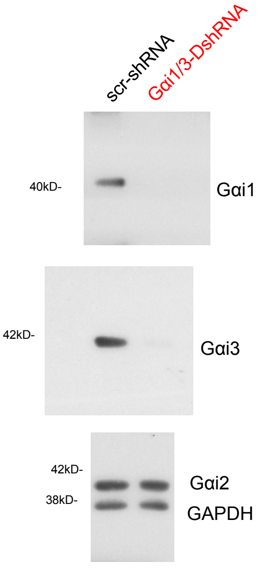

Figure 6

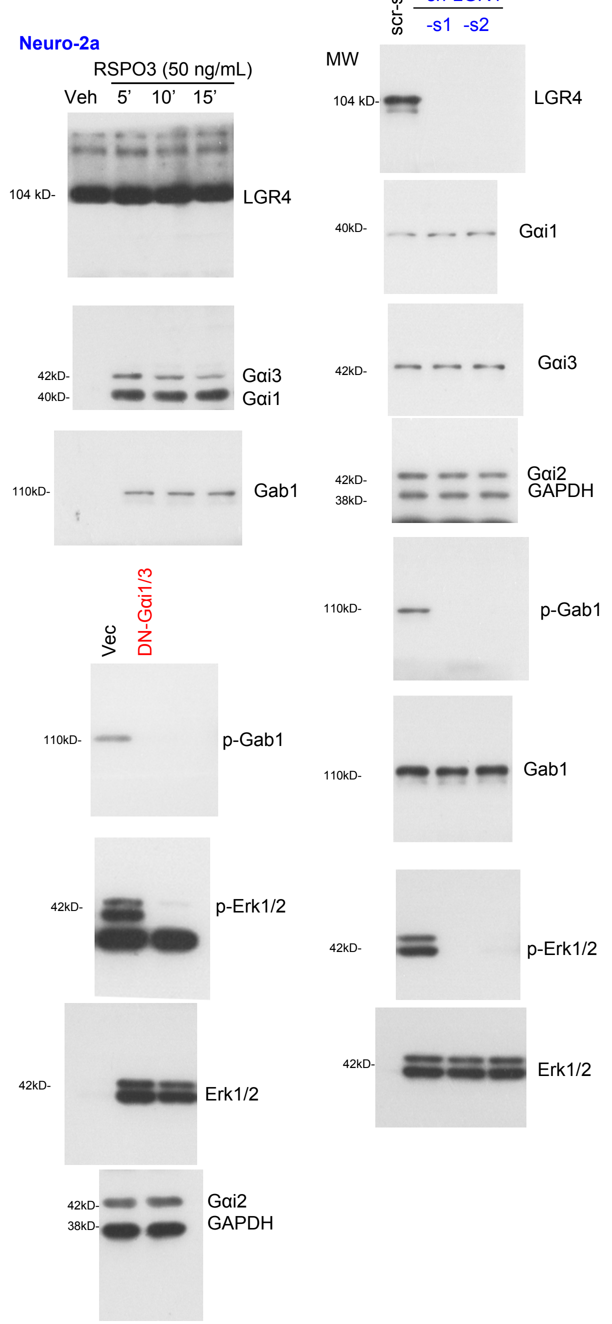

Figure S3.

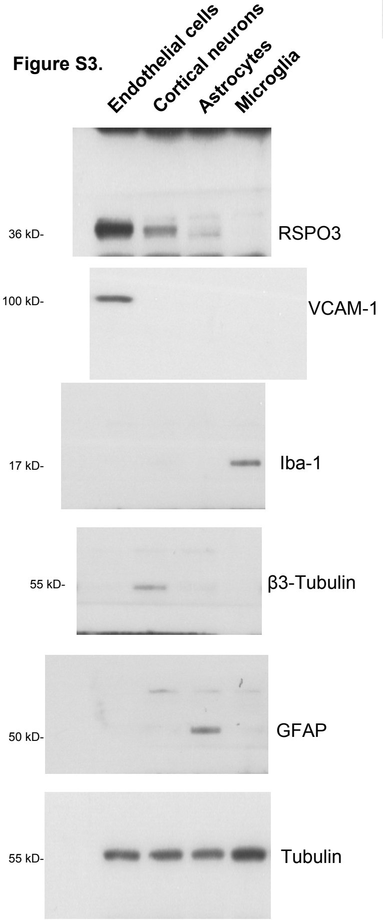

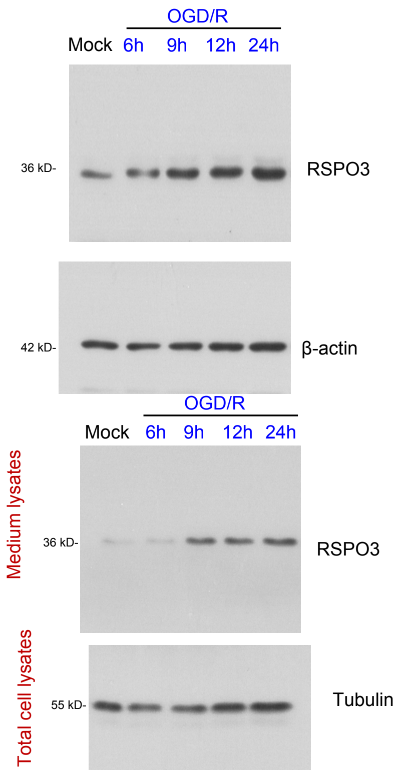

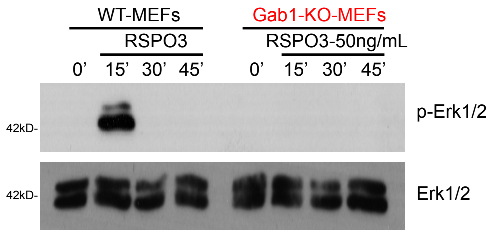

Figure S2.

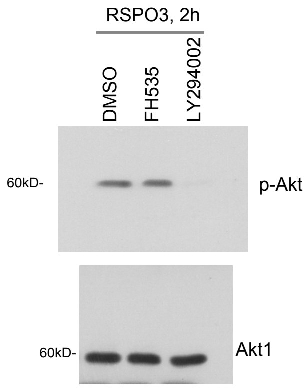

Supplement: Supplementary file 2 — Original Data File [file 41419_2023_6176_MOESM2_ESM.pdf]
