# Supplementary material for: Endothelial cell-derived RSPO3 activates Gαi1/3-Erk signaling and protects neurons from ischemia/reperfusion injury
Source: Cell Death Dis. 2023 Oct 7;14(10):654. doi: 10.1038/s41419-023-06176-2 (PMC10560285; doi:10.1038/s41419-023-06176-2)
Supplement: Supplementary file 4 — Author contribution [file 41419_2023_6176_MOESM4_ESM.pdf]

DECLARATION OF CONTRIBUTIONS TO ARTICLE

ADMC

Manuscript Number: CDDIS-23-1219R

Journal Name: *Cell Death & Disease* (the 'Journal')

Proposed Title of the Contribution:

Endothelial cell-derived RSPO3 activates Gαi1/3-Erk signaling and protects neurons from ischemia/reperfusion injury (the 'Contribution)

Author(s):

Ting-tao Liu, Xin Shi, Hong-wei Hu, Ju-ping Chen, Qin Jiang, Yun-Fang Zhen, Cong Cao, Xue-wu Liu and Jian-gang Liu (the 'Authors')

For all *CDDis* articles, each person named as an author in the published version must be able to show he or she has contributed substantially to the article.

Authorship credit should be based on 1) substantial contributions to conception and design, acquisition of data, or analysis and interpretation of data; 2) drafting the article or revising it critically for important intellectual content; and 3) final approval of the version to be published. Authors should meet conditions 1, 2 and 3.

Any person who cannot be shown to have made a substantial contribution to the article cannot be listed as an author in the final version. The name of any person who is deemed to have made a minor contribution can, however, appear in the Acknowledgments section of the article.

Please complete the table below to indicate the contributions of all named authors to the manuscript.

Author Full Name:	Specification of Contribution to the Manuscript:
Ting-tao Liu	Culture primary neurons and neuronal cells, testing signalings, performed the in vivo animal studies, drafting the article and revising it, with final approval of the version submitted to the journal.
Xin Shi	Culture primary neurons and neuronal cells, testing signalings, performed the in vivo animal studies, drafting the article and revising it, with final approval of the version submitted to the journal.
Hong-wei Hu	Culture primary neurons and neuronal cells, testing signalings, performed the in vivo animal studies, drafting the article and revising it, with final approval of the version submitted to the journal.
Ju-ping Chen	Culture primary neurons and neuronal cells, testing signalings, performed the in vivo animal studies, drafting the article and revising it, with final approval of the version submitted to the journal.
Qin Jiang	Study conception and design, and data analysis, Figure organization, involved in drafting the article and revising it critically for important intellectual content, and with final approval of the version submitted to the journal.
Yun-Fang Zhen	Study conception and design, and data analysis, Figure organization, involved in drafting the article and revising it critically for important intellectual content, and with final approval of the version submitted to the journal.
Cong Cao	Study conception and design, and data analysis, Figure organization, involved in drafting the article and revising it critically for important intellectual content, and with final approval of the version submitted to the journal.
Xue-wu Liu	Study conception and design, and data analysis, Figure organization, involved in drafting the article and revising it critically for important intellectual content, and with final approval of the version submitted to the journal.
Jian-gang Liu	Study conception and design, and data analysis, Figure organization, involved in drafting the article and revising it critically for important intellectual content, and with final approval of the version submitted to the journal.

Please complete the table below to indicate the contributions of all named authors to the figures.

Figure 1:

TL, XS, HH, JC, QJ, CC, XL, YZ and JL

Figure 2:

TL, XS, HH, JC, QJ, CC, XL YZ and JL

Figure 3:

TL, XS, HH, JC, QJ, CC, XL YZ and JL

Figure 4:

TL, XS, HH, JC, QJ, CC, XL and JL

Figure 5:

TL, XS, HH, JC, QJ, CC, XL YZ and JL

Figure 6:

TL, XS, HH, JC, QJ, CC, XL and JL
Figure 7-10,
TL, XS, HH, JC, QJ, CC, XL YZ and JL

Signed for and on behalf of the Author(s):	Print Name:	Date:
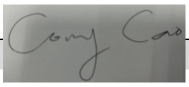	Cong Cao	06/08/2023
